# Gut microbiota and metabolic dysregulation in polycystic ovary syndrome: effects of acupuncture as an adjunct to *in vitro* fertilization on gut dysbiosis, metabolism, and oocyte quality

**DOI:** 10.3389/fmicb.2025.1730714

**Published:** 2026-01-21

**Authors:** Jia-jia Liu, Han Yang, Zhi-yong Xiao, Jie-hui Xie, Lan Su, Yi-ting Li, Xiao-yan Zheng, Wen-hui Hu, Si-jia Fu, Chao-liang Li, Lei Huang, Si-yi Yu, Zheng Yu, Sha Yang, Jie Yang

**Affiliations:** 1 Acupuncture and Tuina School, Chengdu University of Traditional Chinese Medicine, Chengdu, China; 2 Division of Internal Medicine, Institute of Integrated Traditional Chinese and Western Medicine, West China Hospital, Sichuan University, Chengdu, China; 3 Clinical Research Center for Acupuncture and Moxibustion in Sichuan Province, Sichuan Jinxin Xi’nan Women and Children Hospital, Chengdu, China; 4 The Reproductive Center, Sichuan Jinxin Xi’nan Women and Children Hospital, Chengdu, China; 5 Intelligent Medicine School, Chengdu University of Traditional Chinese Medicine, Chengdu, China; 6 Acupoint Effects Key Laboratory of Sichuan Province, Chengdu, China

**Keywords:** acupuncture, follicular fluid, gut microbiota, metabolic, polycystic ovary syndrome

## Abstract

**Introduction:**

Polycystic ovary syndrome (PCOS) is marked by disruptions in metabolic and reproductive endocrine functions. This study synthesizes systemic metabolic profiles, alterations in gut microbiota, and follicular fluid metabolism to elucidate the reproductive and endocrine metabolic changes associated with PCOS. Furthermore, it aims to elucidate the potential mechanisms through which acupuncture may exert therapeutic effects.

**Methods:**

In this open-label randomized controlled trial conducted in China (November 2021–January 2023), 60 women with PCOS scheduled for *In Vitro* Fertilization (IVF) were randomized to receive acupuncture combined with IVF treatment or IVF treatment alone, with 30 healthy women serving as controls. Gut microbiota was sequenced and analyzed by 16S rRNA and metagenomics; follicular fluid metabolites were determined by untargeted metabolomics.

**Results:**

Compared with healthy controls, PCOS exhibited gut microbiota dysbiosis and metabolic disorders. The specific gut microbiota in PCOS dominated by *s_Lachnospiraceae*, *s_Blautia_sp.* and *g_Escherichia-Shigella*, which correlated with body mass index (BMI), waist circumference, waist-to-hip ratio, and hormone levels. Acupuncture combined with IVF significantly regulated glucose and lipid metabolism, reduced *g_Escherichia-Shigell* abundance, and showed potential advantages in enhancing oocyte quality and embryonic developmental potential (*p* = 0.011). Analysis of the correlation between differential metabolites and oocyte and embryo quality demonstrated that methionine sulfoxide and boldione may be key metabolites to affect follicle quality.

**Conclusion:**

PCOS is associated with systemic multi-pathway metabolic dysregulation and gut microbiota dysbiosis. It described the potential therapeutic benefits of acupuncture combined with IVF for PCOS, laying a foundation for further understanding the disease and the mechanisms of acupuncture for PCOS metabolic disorders, and providing directions for future research.

## 1 Introduction

Polycystic ovary syndrome (PCOS) is a prevalent metabolic abnormalities and reproductive disorder characterized by oligo/anovulation, clinical or biochemical hyperandrogenism, and polycystic ovary morphology (Rotterdam ESHRE/ASRM-Sponsored PCOS Consensus Workshop Group, 2004; Zhang et al., 2025). It impacts 6 to 20% of reproductive aged women globally (Stener-Victorin et al., 2024; Vatier and Christin-Maitre, 2024) and is one of the primary causes of infertility (Liu et al., 2024).

PCOS who have failed to conceive after attempting ovulation induction, or with other infertility factors like tubal blockage, often seek assisted reproductive technologies such as *In Vitro* Fertilization-Embryo Transfer (IVF-ET) to achieve pregnancy (Teede et al., 2023b). However, PCOS are accompanied by metabolic abnormalities such as hyperandrogenism, insulin resistance (IR), dyslipidemia and obesity commonly (Glueck and Goldenberg, 2019; Rudnicka et al., 2021; Muscogiuri et al., 2022; Su et al., 2025)^.^ During IVF-ET treatment, metabolic disturbances often lead to widespread pathological phenomena such as chronic inflammation, oxidative stress, and mitochondrial dysfunction (Zhang et al., 2019a; Deng et al., 2024), which may disrupt the microenvironment of follicular development to decrease oocyte maturation rates (Abbott et al., 2006; Jiang et al., 2023; Xiang et al., 2023) and embryo quality (Patel and Carr, 2008) in PCOS. Whether it is possible to find a complementary alternative therapy to regulate metabolic disturbances and improve oocyte quality in PCOS undergoing IVF, is a significant potential direction in the clinical applications of acupuncture-assisted reproductive.

Acupuncture as a significant component of complementary alternative therapies, is widely used in the treatment of gynecological and reproductive diseases (Yu et al., 2020; Li et al., 2023b; Tian et al., 2024; Zhou et al., 2024). Previous studies have shown that acupuncture regulate the hypothalamic–pituitary-ovarian axis (Su et al., 2019), improve follicular development (Chen et al., 2023) and oocyte quality (Budihastuti et al., 2019). However, the specific mechanism is not clear resulting in limited clinical application of acupuncture in the treatment of PCOS.

Gut microbiota inhabit the human gastrointestinal tract and assist the host to exert various physiological and biochemical functions (Human Microbiome Project Consortium, 2012). Imbalance of the gut microbiota is closely related to the pathogenesis of PCOS (Qi et al., 2021), can aggravate metabolic disturbances such as IR, hyperandrogenism and chronic inflammatory status in PCOS (Qi et al., 2019; Li et al., 2023c). Follicular fluid (FF) is mainly composed of plasma exudates and secretions from granulosa and theca cells including proteins, steroids, metabolites and polysaccharides, which facilitate oocyte growth and development (Ambekar et al., 2013; Da Broi et al., 2018). Some studies indicated that gut microbiota influence follicular development and oocyte quality (Li et al., 2023a; Xu et al., 2023), even on ovarian aging (Huang et al., 2024). And changes in the gut microbiome during in IVF treatment are correlated with ovarian response (Fo et al., 2024). With the deepening of the study of gut microbiota and the proposal of the intestine-brain-ovary axis, gut microbiome may improve follicular development and oocyte quality in PCOS by regulating energy metabolism and apoptosis in ovarian granulosa cells (Feng et al., 2022; Luo et al., 2023).

We conducted a randomized controlled clinical study to analyze the characteristics of systemic metabolic and gut microbiota in PCOS. Furthermore, it explores whether acupuncture has a positive effect on oocyte quality through gut microbiota changes and the key metabolic pathways of FF.

## 2 Materials and methods

**Figure 1:**
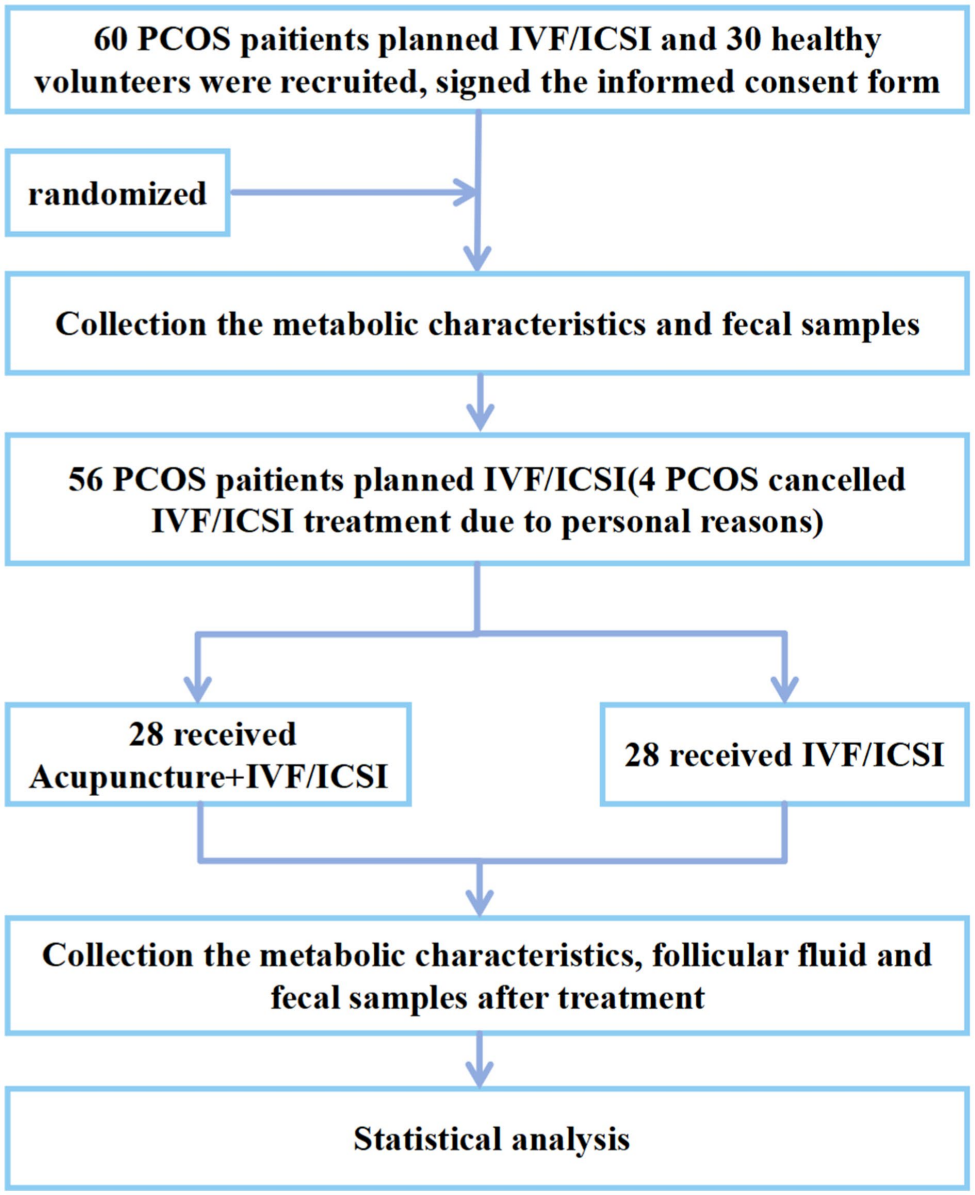
The flowchart of the study.

Our research was divided into three parts and the research flowchart is shown in Figure 1.

### 2.1 Part I: the metabolic characteristics of PCOS

#### 2.1.1 Participant recruitment and randomization

This study was an exploratory investigation into the mechanisms of action. After strict screening according to the inclusion and exclusion criteria (Supplementary Tables 1, 2), 60 PCOS planning to undergo IVF/intracytoplasmic sperm injection (ICSI) from November 2021 to January 2023 at Sichuan Jinxin Xi’nan Women and Children Hospital (former Chengdu Xi’nan Gynecological Hospital), and 30 healthy volunteers as healthy control (HC) group were recruited from the students of Chengdu University of Traditional Chinese Medicine and employees of the hospital mentioned above.

60 PCOS assigned randomly to the acupuncture + IVF/ICSI group (*n* = 30) or IVF/ICSI group (*n* = 30) using number table generated by SPSS 27.0. Each random number was kept in a sealed, opaque envelope by an independent investigator. When eligible participants were enrolled and voluntarily sign the informed consent form, the acupuncturist contacted the independent investigator, who opened the envelope in the numbering order on the envelope, checked the randomized group and informed the acupuncturist, who arranged the corresponding treatment for the participant. Due to the study design, blinding was not feasible. However, strict separation of personnel was implemented, with the treating physicians, outcome assessors, and statisticians remaining independent throughout the study. Both the outcome assessors and the statistician were kept blinded to group allocation until completion of the entire study. In each group, two participants cancelled IVF treatment for personal reasons, 56 PCOS participants were finally completed treatments. The study adhered to the Consolidated Standards of Reporting Trials (CONSORT) and Standards for Reporting Interventions in Controlled Trials of Acupuncture (STRICTA) for reporting randomized trials.

The study protocol has received approval from the Ethics Committee of Sichuan Jinxin Xi’nan Women and Children Hospital (number 2021026) and has been registered with the Chinese Clinical Trial Registry (ChiCTR2200060771). Each participant signed an informed consent form before participating in the study.

#### 2.1.2 Collection the fecal samples and metabolic characteristics at first time

On the fifth day of the menstrual cycle 1 month before IVF treatment, fecal samples were collected in the morning after overnight fasting from PCOS participants. Similarly, healthy participants provided fecal samples on the fifth day of their menstrual cycle in the morning after overnight fasting. Participants were instructed to follow a bland diet for 2 days prior to sampling and to avoid foods and medications containing probiotics or prebiotics. After collection, the samples were briefly stored at room temperature in fecal sample storage tubes and then rapidly transferred to an ultra-low temperature freezer at −80 °C within 2 h waiting to be detected by gut microbiota.

Both groups collected the metabolic characteristics and completed questionnaires on the same day after fecal collection. The metabolic indicators include obesity-related measures including body weight, body mass index (BMI), waist circumference, hip circumference and waist-to-hip ratio (WHR), glucose metabolism-related measures including fasting blood glucose (FBG), fasting insulin (FINS), and homeostatic model assessment of insulin resistance (HOMA-IR = FBG × FINS / 22.5), lipid metabolism-related measures including total cholesterol (TCH), triglyceride (TG), high-density lipoprotein cholesterol (HDLC), low-density lipoprotein cholesterol (LDLC), apolipoprotein A1 (APOA1), apolipoprotein B (APOB), and total testosterone (T).

### 2.2 Part II: the effects of acupuncture combined with IVF treatment on metabolic characteristics and gut microbiota in PCOS

#### 2.2.1 Interventions

Acupuncture + IVF/ ICSI group (A group) and IVF/ ICSI group (B group) received the same IVF/ICSI treatment and the same lifestyle guidance (Balen et al., 2016), with the A group receiving additional acupuncture treatment. Treatments and medications during IVF were prescribed by the reproductive physician according to the specific circumstances of PCOS, and all PCOS included underwent controlled ovarian hyperstimulation (COH) using the gonadotropin-releasing hormone antagonist protocol (Bosch et al., 2020; Chen et al., 2022).

Acupuncture treatment was divided into two phases for a total of 20 sessions. The first phase started on day 5 of the menstrual cycle before a month of COH, three times a week, every other day, 30 min each time, for a total of 12 times. The second phase started on day 3 of COH and received acupuncture treatment once a day for 30 min until the trigger day, for a total of 8 times. The treatment was performed by an acupuncturist who had a certificate of qualification as a licensed physician and accepted training in a standardized procedure. A disposable acupuncture needles (φ0.25 × 25 mm, φ0.25 × 40 mm, φ0.25 × 50 mm, and φ0.25 × 75 mm, Hwato, Suzhou, China) was vertically or diagonally inserted into each acupoint to 25–75 mm to obtain a deqi sensation (a distention, soreness, heaviness or numbness sensation), and the needles was performed once to maintain the deqi state at the 15th min of needle retention. The treatment acupoints were alternated between the two groups, with group 1 in the supine position, including GV20 (Baihui), GV 24 (Shenting), GB 13 (Benshen), CV12 (Zhongwan), CV 6 (Qihai), CV4 (Guanyuan), ST25 (Tianshu), GB26 (Daimai), KI12 (Dahe), EX-CA1 (Zigong), ST36 (Zusanli), ST40 (Fenglong), and LR3 (Taichong), and group 2 in the prone position, including BL23 (Shenshu), BL25 (Dachangshu), BL32 (Ciliao), BL53 (Zhibian), SP9 (Yinlingquan), SP 6 (Sanyinjiao), and KI3 (Taixi) (Supplementary Figure 1; Supplementary Table 3).

#### 2.2.2 Collection the clinical outcomes, the second time metabolic characteristics and fecal samples after interventions

The primary outcome was oocyte maturation rate, and secondary outcomes included Follicle-to-Oocyte Index rate, embryo quality (including normal fertilization rate, the rate of high-quality embryos, the rate of high-quality embryos at cleavage stage, the rate of high-quality embryos at blastocyst stage, the rate of available embryos, blastocyst formation rate). The standard for high-quality embryos was shown in Supplementary Tables 4–7.

Fecal samples were collected from PCOS participants in the morning of the oocyte retrieval day for gut microbiota detection (collection standards in 2.1.2). After collection, participants were collected blood sampling for metabolic characteristic and completed questionnaire.

### 2.3 Part III: the effects of acupuncture combined with IVF treatment on the quality of follicles, embryos and follicular fluid metabolism in PCOS

This part evaluated the clinical efficacy and metabolic effect of acupuncture combined with IVF on PCOS undergoing IVF from the perspectives of oocyte quality, embryo quality, and follicular fluid metabolism.

The FF was collected on the day of oocyte retrieval from PCOS. FF in the first and last tube may affect the experimental results due to mixing organic solvent rinse. Therefore, yellowish, clear, and bloodless FF were collected after removing the first and last FF mixed thoroughly, and centrifuged at 12,000 rpm, temperature of 4 °C for 10 min. Supernatants were then collected and frozen in an ultra-low temperature freezer at −80 °C waiting to be metabolite extracted.

### 2.4 16S rRNA gene sequencing and analysis

16S rRNA gene sequencing was carried out on all fecal samples, and metagenomic sequencing was implemented on a randomly chosen subset that constituted one-tenth of the entire sample. Total genomic DNA was extracted from fecal samples using the SDS method, with concentration and purity assessed via 1% agarose gels electrophoresis. DNA was diluted to 1 ng/μL in sterile water based on measured concentrations. The V3-V4 regions of the 16S rRNA gene were amplified using specific primers 341F (5′-CCTAYGGGRBGCASCAG-3′) and 806R (5′-GGACTACNNGGGTATCTAAT-3′) (Qin et al., 2024). The PCR amplifications utilized Phusion® High-Fidelity PCR Master Mix, with 10 ng of template DNA input. PCR products were quantified and qualified by mixing equal volumes with 1X loading buffer containing SYBR Green and running electrophoresis on 2% agarose gel. The resulting PCR amplicons were cleaned up using Qiagen gel extraction kits and sequencing libraries were assembled with TruSeq® DNA PCR-free kits, incorporating index codes. The libraries’ quality underwent examination with a Qubit 2.0 fluorometer and the Agilent Bioanalyzer 2,100 system. Finally, libraries were sequenced on the Illumina NovaSeq platform, yielding 250 bp paired-end reads.

The raw data obtained were concatenated and filtered, followed by denoising using DADA2. Sequences with abundances of less than 5 were filtered out to yield the final amplified sequence variants (ASVs). After obtaining the ASVs, alpha diversity and *β* diversity analyses were conducted for the ASVs to provide insights into species richness and evenness within samples, and differences in community structure between different groups (Li et al., 2020). Species exhibiting significant differences between and within groups before and after treatment were screened using the Linear discriminant analysis Effect Size (LEfSe) method.

### 2.5 Metagenomic sequencing and analysis

For library construction, genomic DNA was fragmented into brief segments, followed by end repair, A-tailing, adapter ligation, purification, and PCR amplification. Qualified libraries were pooled based on concentrations and data output, while size distribution was evaluated via bioanalyzer. Then sequenced on Illumina platform with PE150 reads.

DIAMOND software was used to align Unigenes with those in the Kyoto Encyclopedia of Genes and Genomes (KEGG) database. From the alignment results of each sequence, the Best Blast Hit results were selected for subsequent analysis. According to the alignment results, the relative abundance at different functional levels was calculated. Based on the abundance table at each taxonomy level, combined with principal component analysis of dimension reduction, metabolic pathway comparative and LEfSe on inter-group functional difference was analyzed.

### 2.6 Metabolomics sequencing and analysis

The FF samples were centrifuged after using 80% methanol aqueous and mass spectrometric grade water. The supernatant was collected and analyzed using ultra-high performance liquid chromatography combined with high-resolution mass spectrometry, specifically liquid chromatography-mass spectrometry technology for separation. Data files generated by the instrument were imported into CD3.1 search software for processing, and search comparisons were conducted using mzCloud, mzVault, and Masslist databases to ultimately obtain metabolite identification and relative quantification results. For quality control purposes, QC samples were also established during the processing of the samples.

Identified metabolites were annotated using KEGG database. In the multivariate statistical analysis part, metabolomics data processing software metaX was used to transform the data and partial least squares discriminant analysis (PLS-DA) was performed. Stem plots were made using the R package ggplot2 to visualize the data to screen for metabolites. Correlations between differential metabolites (Spearman correlation coefficients) were performed using the corrplot software package in R language to draw correlation plots. Enrichment analysis was performed using the KEGG database to investigate the function and metabolic pathways of metabolites.

### 2.7 Statistical analysis

Statistical analysis was conducted using IBM SPSS Statistics 27.0 software, and chi square test was used for count data; Exploratory analysis was first conducted on the quantitative data. If the data conforms to normal distribution and homogeneity of variance, t-test was used. Among them, inter group comparison was conducted using two independent sample t-test; Two paired sample t-test was used for comparison before and after treatment within the group; The Mann Whitney test was used for data that does not follow a normal distribution. All tests were conducted using a two-sided test, with a statistical threshold set at *p*-value < 0.05. Concurrently, correlation analyses were conducted between key species and clinical parameters to investigate the potential clinical impacts of various species.

## 3 Results

### 3.1 Metabolic characteristics of PCOS compared with HC

#### 3.1.1 Comparison of biochemical indicators between PCOS and HC

**Figure 2:**
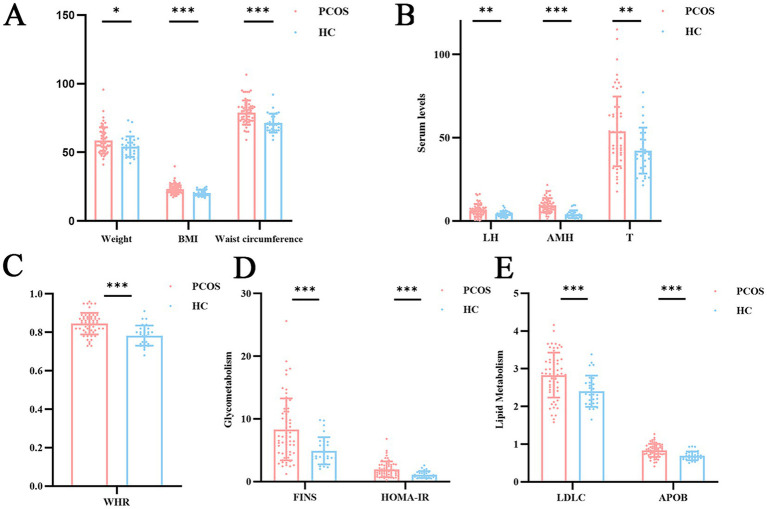
Comparison of abnormal metabolic characteristics between PCOS and HC. **(A)** Anthropometric measurements including body weight, BMI, and waist circumference between PCOS and HC, **(B)** Serum hormone levels including LH, AMH and T between PCOS and HC, **(C)** WHR between PCOS and HC, **(D)** Glycometabolism parameters including FINS and HOMA-IR between PCOS and HC, **(E)** Lipid metabolism indices including LDLC and APOB between PCOS and HC. PCOS, polycystic ovary syndrome; HC, healthy control; BMI, body mass index; LH, luteinizing hormone; AMH, anti-müllerian hormone; T, testosterone; WHR, waist to hip ratio; FINS, fasting insulin; HOMA-IR, homeostatic model assessment of insulin; LDLC, low-density lipoprotein cholesterol; APOB, apolipoprotein B; *p < 0.05; **p < 0.01; ***p < 0.001.

Compared to HC, PCOS had significant differences in weight, BMI, waist circumference, and WHR. There were no significant differences in thyroid function indicators between two groups. In terms of sex hormones and Anti-Müllerian Hormone (AMH) levels, PCOS had notably higher levels of T, luteinizing hormone (LH), and AMH compared to HC. In glucose and lipid metabolism, PCOS showed significantly higher FINS and HOMA-IR, as well as higher levels of LDL-C and APOB, compared to HC (Table 1; Figure 2). Other indicators showed no significant differences.

**Table 1:** Metabolic objective indicators of PCOS and HC.

Characteristics	PCOS (*n* = 60)	HC (*n* = 30)	Absolute difference between groups (95% CI)	*p*-value
Age (years), (mean ± SD)	29.47 ± 3.638	26.80 ± 2.295	2.667 (1.415–3.919)	<0.001***
Weight (kg), M (IQR)	55.750 (12.8)	53.750 (10.6)	4.000 (0.400–7.700)	0.028*
BMI (kg/m^2^), M (IQR)	22.635 (4.598)	19.980 (3.829)	2.292 (1.298–3.626)	<0.001***
Waist circumference (cm), (mean ± SD)	78.930 ± 8.474	71.340 ± 6.865	7.590 (4.044–11.136)	<0.001***
Hip circumference (cm), (mean ± SD)	93.466 ± 5.670	91.000 ± 5.568	2.466(−0.039–4.971)	0.054
WHR, (mean ± SD)	0.843 ± 0.052	0.784 ± 0·052	0.059 (0.036–0.082)	<0.001***
TSH (μIU/ml), M (IQR)	2.110 (1.58)	2.250 (1.98)	−0.380(−0.920–0.130)	0.132
FT4 (pmol/L), M (IQR)	11.475 (2.62)	11.23 (1.76)	0.000(−0.650–0.820)	0.993
TPOAb (IU/ml), M (IQR)	1.050 (2.58)	1.000 (1.95)	0.200(−0.200–0.600)	0.333
Total testosterone (ng/dl), (mean ± SD)	47.155 (24.35)	40.345 (20.40)	9.545 (2.350–16.950)	0.009**
AMH (ng/ml), M (IQR)	8.33 (5.69)	3.465 (2.67)	4.725 (3.340–6.290)	<0.001***
Serum levels				
LH (mIU/ml), M (IQR)	6.345 (4.023)	3.995 (1.697)	1.945 (0.680–3.190)	0.002**
FSH (mIU/ml), M (IQR)	5.840 (2.29)	5.740 (1.77)	−0.290(−1.020–0.390)	0.409
PRL (uIU/ml), M (IQR)	247.45 (175.19)	263.710 (233.42)	−43.520(−97.960–14.300)	0.150
E2 (pg/ml), M (IQR)	28.575 (15.75)	27.000 (13.250)	2.000(−3.000–7.000)	0.406
P (ng/ml), M (IQR)	0.450 (0.35)	0.485 (0.25)	0.000(−0.110–0.110)	0.966
Glycometabolism				
FBG (mmol/L), (mean ± SD)	5.241 ± 0.345	5.092 ± 0.323	0.150(−0.000–0.300)	0.050
FINS (mIU/L), M (IQR)	7.215 (6.55)	4.040 (2.75)	2.720 (1.220–4.280)	<0.001***
HOMA-IR, M (IQR)	1.635 (1.643)	0.905 (0.682)	0.676 (0.283–1.054)	<0.001***
Lipid Metabolism				
TCH (mmol/L), (mean ± SD)	4.469 ± 0.674	4.315 ± 0.564	0.154(−0.131–0.438)	0.286
TG (mmol/L), M (IQR)	1.445 (0.96)	1.19 (0.42)	0.190(−0.080–0.430)	0.192
HDLC (mmol/L), M (IQR)	1.185 (0.41)	1.260 (0.42)	−0.100(−0.220–0.020)	0.093
LDLC (mmol/L), (mean ± SD)	2.842 ± 0.579	2.404 ± 0.415	0.439 (0.203–0.675)	<0.001***
APOA1 (g/L), M (IQR)	1.315 (0.33)	1.240 (0.44)	0.010(−0.120–0.130)	0.797
APOB (g/L), (mean ± SD)	0.836 ± 0.171	0.690 ± 0.116	0.146 (0.077–0.215)	<0.001***

BMI, body mass index; WHR, waist to hip ratio; TSH, thyroid stimulating hormone; FT4, free Thyroxine; TPOAb, thyroid peroxidase antibody; AMH, anti-müllerian hormone; LH, luteinizing hormone; FSH, follicle stimulating hormone; PRL, prolactin; E2, estradiol; P, progesterone; FBG, fasting blood glucose; FINS, fasting insulin; HOMA-IR, homeostatic model assessment of insulin; TCH, total cholesterol; TG, triglyceride; HDLC, high-density lipoprotein cholesterol; LDLC, low-density lipoprotein cholesterol; APOA1, apolipoprotein A1; APOB, Apolipoprotein B. *p < 0.05, **p < 0.01, ***p < 0.001.

#### 3.1.2 16S rRNA gene sequencing shown the difference of gut microbiota between PCOS and HC

**Figure 3:**
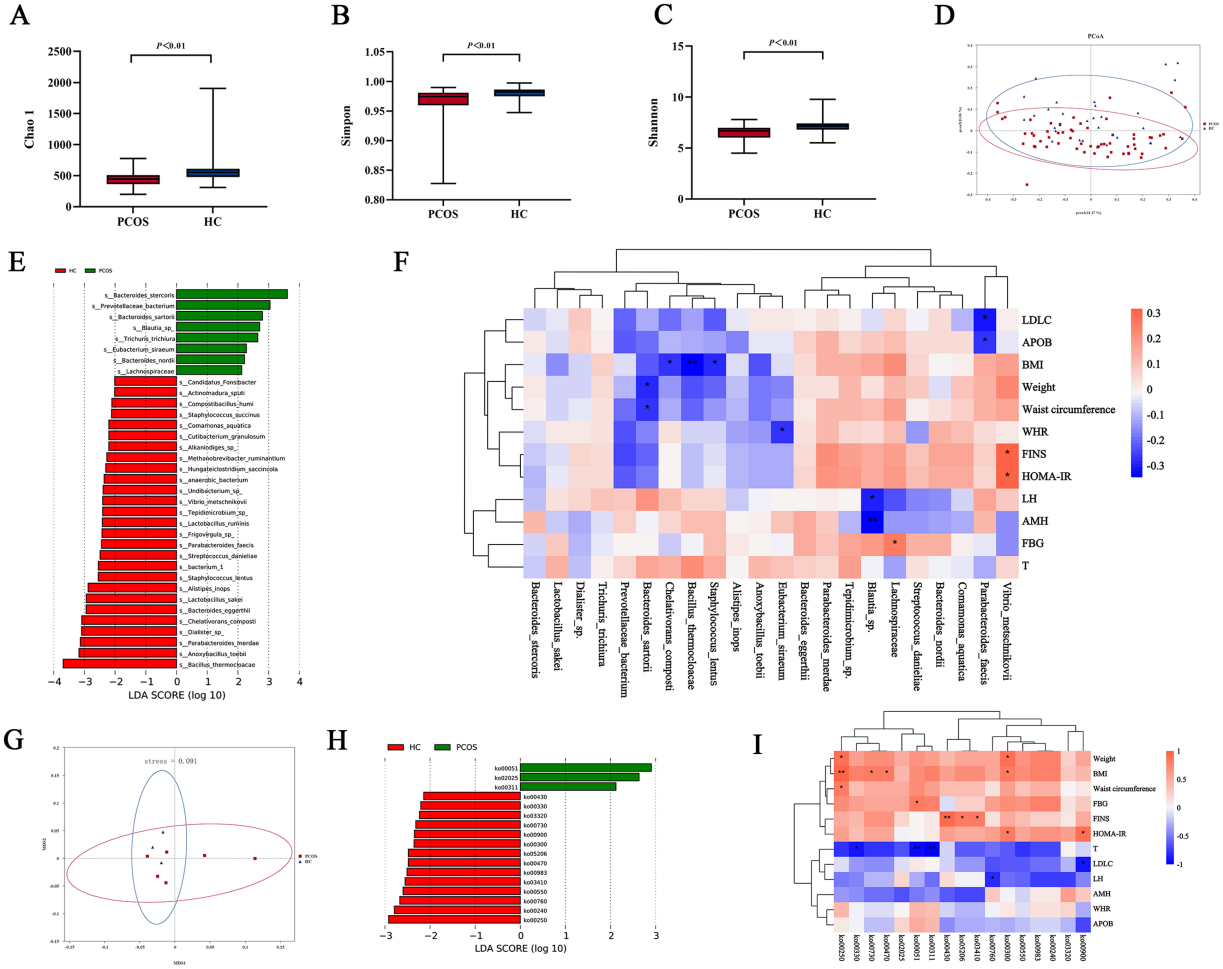
Comparison of gut microbiota between PCOS and HC. **(A–C)** Chao1, Shannon, and Simpson indices between PCOS and HC. **(D)** PCoA based on the weighted_unifrac of gut microbiota between PCOS and HC. **(E)** Differential microbiota at the species level between PCOS and HC. **(F)** The correlation heatmap between differential microbiota and clinical metabolic abnormal indicators in PCOS. **(G)** PCoA in KEGG level 3 metabolic pathways between PCOS and HC. **(H)** Differences in annotated KEGG level 3 metabolic pathways between PCOS and HC. **(I)** The correlation heatmap between differential metabolic pathways and clinical metabolic abnormal indicators. PCOS, polycystic ovary syndrome; HC, healthy control.

A Venn diagram showed that PCOS and HC shared 2,573 ASVs, with 4,474 unique ASVs in HC and 4,158 unique ASVs in PCOS, indicating fewer gut microbiota in PCOS compared to HC (Supplementary Figure 2A). Stacked column charts at the phylum, genus, and species levels revealed differences in gut microbiota composition between PCOS and HC (Supplementary Figures 2B–D). Alpha diversity analysis showed that the Chao1 index, Shannon index, and Simpson index were significantly lower in the PCOS group than in HC (*p* < 0.05), indicating lower richness and diversity of gut microbiota in PCOS compared to HC (Figures 3A–C). Principal Coordinate Analysis (PCoA) based on the weighted_unifrac distance demonstrated significant differences in gut microbiota community composition between PCOS and HC (*p* = 0.018) (Figure 3D). The LEfSe analysis compared the differential microbiota between PCOS and HC at species level (Figure 3E). Spearman correlation analysis between differential microbiota and clinical metabolic indicators at the species level revealed that *s_Bacteroides_sartorii* was negatively correlated with weight and waist circumference, BMI was negatively correlated with *s_Chelativorans_composti*, *s_Bacillus_thermocloacae*, and *s_Staphylococcus_lentus*, WHR was negatively correlated with *s_Eubacterium_siraeum*, LH and AMH were negatively correlated with *s_Blautia_*sp., FBG was positively correlated with *s_Lachnospiraceae*, *s_Parabacteroides_faecis* was negatively correlated with LDLC and APOB, and *s_Vibrio_metschnikovii* was positively correlated with FINS and HOMA-IR (Figure 3F).

#### 3.1.3 Metagenomic sequencing shown the difference of metabolic pathways between PCOS and HC

After metagenomic sequencing and KEGG annotation, we found that PCOS and HC showed significant differences in metabolic pathways based on the PCoA plot (Figure 3G). The LEfSe analysis compared the differential metabolic pathways between PCOS and HC at KEGG level 3 (Figure 3H). Compare with HC, PCOS exhibited a significant increase in fructose metabolism (ko00051), which was significantly correlated with fasting blood glucose. HC had higher levels of metabolism (ko00760), pyrimidine metabolism (ko00240), and amino acid metabolism (ko00250) compared to PCOS. Additionally, amino acid metabolism (ko00250) was significantly correlated with weight, BMI, and waist circumference (Figure 3I).

### 3.2 Acupuncture combined with IVF treatment decreased the relative abundance of *g_Escherichia-Shigella* and improved systemic glucose and lipid metabolism disorders in PCOS

#### 3.2.1 Metabolic characteristics changes

There was no difference in baseline between A group and B group (Supplementary Table 8). After acupuncture combined with IVF treatment, the changes in body weight, BMI, and TCH were greater than IVF treatment only (Table 2). In comparison within A group before and after treatment, T and TG increased, while TCH, LDL-C, APOB, and FBG decreased (Table 3). In comparison within B group before and after treatment, body weight, BMI, hip circumference, T, TG, APOA1, and FINS increased, while TCH, LDL-C, and APOB decreased (Table 4).

**Table 2:** Comparison of changes in clinical variables after treatment between two groups.

Secondary outcomes	Difference in treatment before and after A group (*n* = 28)	Difference in treatment before and after B group (*n* = 28)	Absolute difference between groups (95% CI)	*p*-value
Weight (kg), (mean ± SD)	0.579 ± 1.814	−0.629 ± 1.332	1.207 (0.354, 2.060)	0.006 **
BMI (kg/m^2^), M (IQR)	0.090 (0.77)	−0.230 (0.50)	0.410 (0.060, 0.750)	0.007 **
Waist circumference (cm), M (IQR)	0.000 (5.13)	0.000 (3.88)	0.000(−2.000, 2.000)	0.799
Hip circumference (cm), (mean ± SD)	0.428 ± 3.828	−0.900 ± 2.011	1.328(−0.323, 2.979)	0.112
WHR, (mean ± SD)	−0.005 ± 0.05	0.000 ± 0.05	−0.010(−0.030, 0.020)	0.468
Total testosterone (ng/dl), (mean ± SD)	−46.756 ± 35.146	−50.733 ± 29.959	3.977(−13.521, 21.474)	0.650
TCH (mmol/L), (mean ± SD)	0.563 ± 0.467	0.256 ± 0.461	0.307 (0.058, 0.556)	0.017 *
TG (mmol/L), (mean ± SD)	−0.408 ± 0.609	−0.393 ± 0.593	−0.015(−0.337, 0.307)	0.926
HDL-C (mmol/L), M (IQR)	0.045 (0.29)	0.000 (0.16)	0.070(−0.010, 0.170)	0.110
LDL-C (mmol/L), (mean ± SD)	0.688 ± 0.545	0.476 ± 0.469	0.213(−0.060, 0.485)	0.124
APOB (g/L), (mean ± SD)	0.118 ± 0.139	0.115 ± 0.159	0.003(−0.077, 0.083)	0.943
APOA1 (g/L), M (IQR)	−0.110 (0.31)	−0.135 (0.26)	0.050(−0.060, 0.170)	0.471
FBG (mmol/L), M (IQR)	0.295 (0.63)	0.130 (0.56)	0.160(−0.060, 0.370)	0.140
FINS (mIU/L), M (IQR)	−0.490 (5.05)	−1.120 (4.73)	1.395(−0.620, 3.520)	0.128
HOMA-IR, M (IQR)	0.050 (1.05)	−0.270 (1.27)	0.345(−0.130, 0.840)	0.145

*p < 0.05, **p < 0.01, ***p < 0.001.

**Table 3:** Comparison of changes in clinical variables before and after treatment in Group A.

Secondary outcomes	Apre (*n* = 28)	Apost (*n* = 28)	Absolute difference in group (95% CI)	*p*-value
Weight (Kg), (mean ± SD)	58.518 ± 7.749	57.939 ± 7.570	0.579(−0.125, 1.282)	0.103
BMI (kg/m^2^), M (IQR)	22.875 (4.49)	22.530 (3.89)	−0.200(−0.415, 0.040)	0.154
Waist circumference (cm), (mean ± SD)	79.323 ± 7.186	79.296 ± 7.714	0.026(−1.617, 1.670)	0.974
Hip circumference (cm), (mean ± SD)	93.893 ± 5.105	93.464 ± 4.872	0.428(−1.056, 1.913)	0.559
WHR, (mean ± SD)	0.843 ± 0.047	0.847 ± 0.052	−0.004(−0.022, 0.014)	0.651
Total testosterone (ng/dl), (mean ± SD)	55.956 ± 24.824	102.713 ± 38.624	−46.756(−60.385, −33.128)	<0.001***
TCH (mmol/L), (mean ± SD)	4.628 ± 0.682	4.066 ± 0.552	0.563 (0.381, 0.744)	<0.001***
TG (mmol/L), (mean ± SD)	1.507 ± 0.745	1.915 ± 0.788	−0.408(−0.644, −0.172)	0.001**
HDL-C (mmol/L), (mean ± SD)	1.306 ± 0.297	1.230 ± 0.301	0.076(−0.011, 0.162)	0.085
LDL-C (mmol/L), (mean ± SD)	2.974 ± 0.566	2.286 ± 0.600	0.688 (0.476, 0.899)	<0.001***
APOB (g/L), (mean ± SD)	0.861 ± 0.162	0.743 ± 0.157	0.118 (0.064, 0.172)	<0.001***
APOA1 (g/L), (mean ± SD)	1.419 ± 0.273	1.517 ± 0.338	−0.099(−0.210, 0.013)	0.080
FBG (mmol/L), M (IQR)	5.195 (0.49)	5.080 (0.53)	−0.290(−0.465, −0.090)	0.007**
FINS (mIU/L), M (IQR)	6.435 (6.56)	7.010 (4.10)	0.070(−1.885, 1.420)	0.952
HOMA-IR, M (IQR)	1.560 (1.58)	1.530 (1.04)	−0.125(−0.525, 0.200)	0.456

*p < 0.05, **p < 0.01, ***p < 0.001.

**Table 4:** Comparison of changes in clinical variables before and after treatment in Group B.

Secondary outcomes	Bpre (*n* = 28)	Bpost (*n* = 28)	Absolute difference in group (95% CI)	*p*-value
Weight (Kg), (mean ± SD)	58.054 ± 11.374	59.682 ± 11.422	−0.629(−1.145, −0.112)	0.019*
BMI, (mean ± SD)	23.556 ± 4.471	23.809 ± 4.491	−0.253(−0.459, −0.047)	0.018*
Waist circumference, (mean ± SD)	79.000 (12.10)	77.750 (10.8)	0.000(−1.250, 1.250)	0.948
Hip circumference, (mean ± SD)	93.329 ± 6.527	94.229 ± 6.762	−0.900(−1.680, −0.120)	0.025*
WHR, M (IQR)	0.855 (0.07)	0.835 (0.08)	0.000(−0.025, 0.010)	0.468
Total testosterone (ng/dl), (mean ± SD)	52.875 ± 17.861	103.608 ± 34.628	−50.733(−62.350, −39.117)	<0.001***
TCH (mmol/L), (mean ± SD)	4.325 ± 0.551	4.070 ± 0.520	0.256 (0.077, 0.435)	0.007**
TG (mmol/L), (mean ± SD)	1.546 ± 0.771	1.940 ± 0.621	−0.393(−0.623, −0.163)	0.002**
HDL-C (mmol/L), M (IQR)	1.125 (0.25)	1.160 (0.31)	0.005(−0.050, 0.050)	0.829
LDL-C (mmol/L), (mean ± SD)	2.746 ± 0.546	2.271 ± 0.556	0.475 (0.293, 0.657)	<0.001***
APOB (g/L), (mean ± SD)	0.826 ± 0.170	0.711 ± 0.206	0.115 (0.053, 0.177)	0.001**
APOA1 (g/L), M (IQR)	1.245 (0.35)	1.470 (0.48)	0.140 (0.070, 0.275)	0.001**
FBG (mmol/L), (mean ± SD)	5.199 ± 0.344	5.071 ± 0.369	0.128(−0.010, 0.266)	0.067
FINS (mIU/L), (mean ± SD)	9.276 ± 4.761	10.792 ± 5.873	−1.516(−2.793, −0.239)	0.022*
HOMA-IR, (mean ± SD)	2.170 ± 1.158	2.373 ± 1.497	−0.203(−0.551, 0.146)	0.243

*p < 0.05, **p < 0.01, ***p < 0.001.

#### 3.2.2 Gut microbiota changes based on 16S rRNA gene sequencing

**Figure 4:**
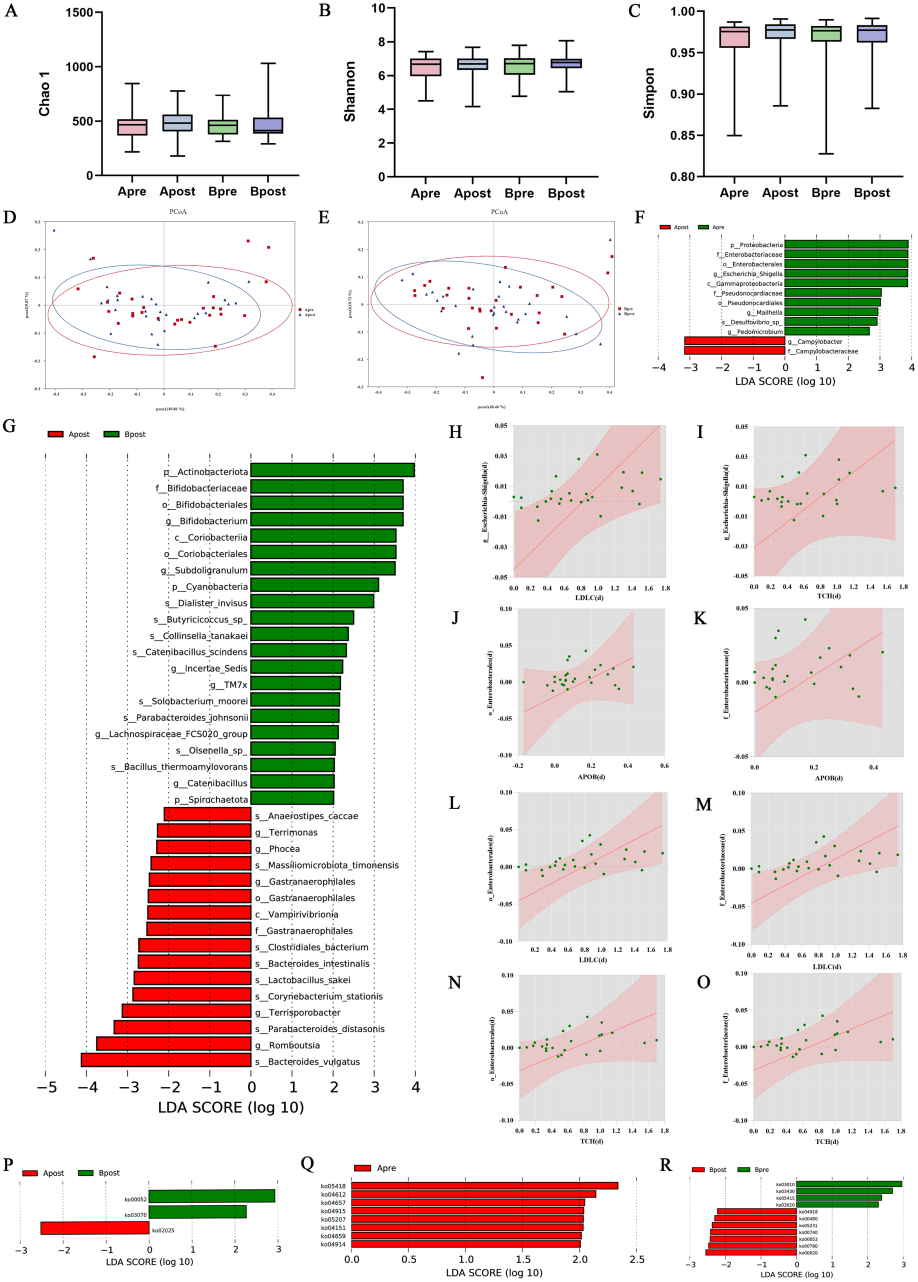
Comparison of gut microbiota between A group and B group. **(A–C)** Changes in Chao1, Shannon, and Simpson indices before and after treatment in A group and B group. **(D)** PCoA based on the weighted_unifrac of gut microbiota before and after treatment in A group. **(E)** PCoA based on the weighted_unifrac of gut microbiota before and after treatment in B group. **(F)** Differential microbiota before and after treatment in A group. **(G)** Comparison of differential microbiota after treatment between A group and B group. **(H–O)** Correlation analysis of *g_Escherichia-Shigella*, *o_Enterobacterales*, *f_Enterobacteriaceae* with LDLC, TCH, and APOB. **(P)** Differences in KEGG level 3 metabolic pathways after treatment between the two groups. **(Q)** Differences in KEGG level 3 metabolic pathways before and after treatment in A group. **(R)** Differences in KEGG level 3 metabolic pathways before and after treatment in B group. Apre, before acupuncture + IVF treatment; Apost, after acupuncture + IVF treatment; Bpre, before IVF treatment; Bpost, after IVF treatment.

The composition of the microbiota between A group and B group before and after treatment showed in Supplementary Figures 3A–C. There were no significant changes in *α* diversity and *β* diversity between the A group and the B group after treatment (Figures 4A–E). Through LEfSe analysis, the abundance of *s_Lactobacillus_sakei* in the A group was significantly higher than that in the B group after acupuncture combined with IVF treatment (Figure 4F). However, compared to PCOS, *s_Lactobacillus_sakei* was more abundant in HC at part I. There were significant differences in the abundance of multiple bacterial species before and after treatment after acupuncture combined with IVF treatment, while showed no significant changes after IVF treatment (Figure 4G). Spearman analysis found that *g_Escherichia-Shigella* in the A group was positively correlated with LDLC and TCH, and the relative abundance of *o_Enterobacterales* before and after treatment was positively correlated with APOB, TCH, and LDLC. The relative abundance of *f_Enterobacteriaceae* before and after treatment was positively correlated with APOB, TCH, and LDLC (Figures 4H–O).

#### 3.2.3 Changes in metabolic pathways based on metagenomic sequencing

After using metagenomic sequencing and LEfSe analysis KEGG level 3 metabolic pathways, it was found that the galactose metabolism pathway (ko00052) and bacterial secretion system metabolism pathway (ko03070) decreased in A group compared to B group (Figure 4P). After acupuncture combined with IVF treatment, the antigen processing and presentation (ko04612), IL-17 signaling pathway (ko04657), estrogen signaling pathway (ko04915), chemical carcinogen receptor activation (ko05207), PI3K-Akt signaling pathway (ko04151), Th17 cell differentiation (ko04659), and progesterone-mediated oocyte maturation (ko04914) decreased (Figure 4Q). These pathways mainly focused on oocyte maturation and inflammatory response. After IVF treatment, DNA mismatch repair (ko03430), diabetic cardiomyopathy (ko05415), RNA polymerase (ko03020) decreased, while metabolic pathways such as glutathione metabolism (ko00480), cancer choline metabolism (ko05231), and riboflavin metabolism (ko00740) increased (Figure 4R).

### 3.3 Effects of acupuncture combined with IVF on the quality of oocyte and the metabolism of follicular fluid in PCOS

#### 3.3.1 The evaluation of oocytes and embryos

**Figure 5:**
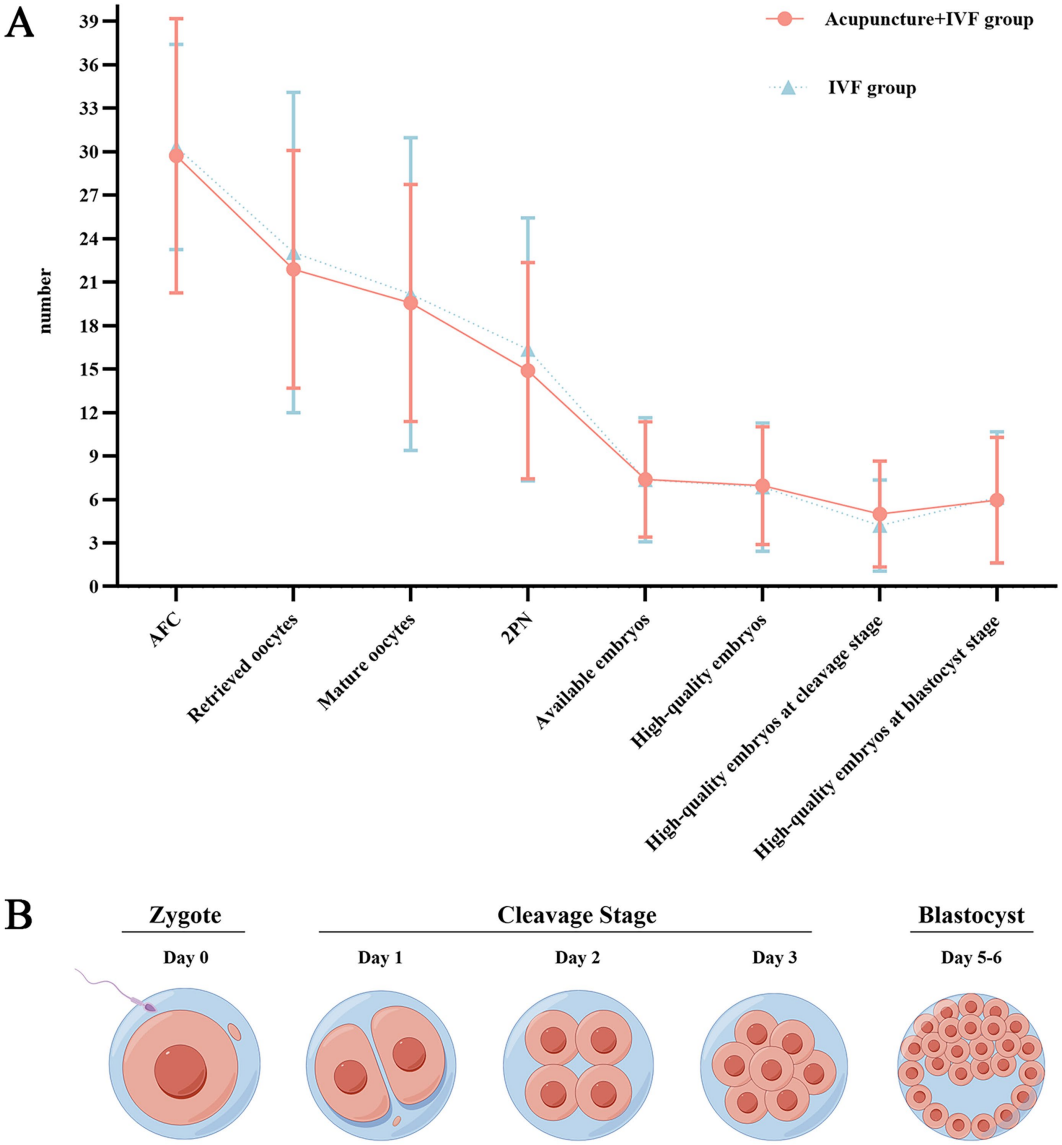
**(A)** Comparison of oocyte and embryo quality between two groups. **(B)** The process of early embryonic development during IVF.

The normal fertilization rate of A group was significantly lower than B group, possibly due to the higher number of oocytes retrieved in B group, but the rate of high-quality embryos at the cleavage stage in A group was significantly higher than B group, indicating that although the number of oocytes retrieved in the B group was higher, and the potential for later development was insufficient (Table 5; Figures 5A,B).

**Table 5:** Oocyte and embryo quality outcomes.

Outcomes, *n*/*N* (%)	A group (*n* = 28)	B group (*n* = 28)	Absolute difference in group (95% CI)	*p*-value
Oocyte maturation rate	548/613 (89.4%)	565/645 (87.6%)	1.80% (−1.8, 5.3%)	0.318
IVF,25VS 25	480/539 (89.1%)	510/580 (87.9%)	1.2% (−2.7, 4.9%)	0.557
ICSI,3 VS 3	68/74 (91.9%)	55/65 (84.6%)	7.3% (−3.6, 18.8%)	0.180
FOI	613/832 (73.7%)	645/849 (76.0%)	−2.3% (−1.9, 6.4%)	0.279
Embryo quality outcomes				
Normal fertilization rate	76.1% (417/548)	81.1% (458/565)	−5.0% (0.2, 9.8%)	0.043
The rate of high-quality embryos	46.8% (195/417)	41.9% (192/458)	4.8% (−1.7, 11.4%)	0.150
The rate of high-quality embryos at cleavage stage	33.6% (140/417)	25.8% (118/458)	7.8% (1.8, 13.8%)	0.011*
The rate of high-quality embryos at blastocyst stage	40.0% (167/417)	37.6% (172/458)	2.5% (−4.0, 8.9%)	0.450
The rate of available embryos	49.6% (207/417)	45.0% (206/458)	4.7% (−2.0, 11.2%)	0.168
Blastocyst formation rate	41.5% (173/417)	38.6% (177/458)	2.8% (−3.6, 9.3%)	0.392

IVF, in vitro fertilization; ICSI, intracytoplasmic sperm injection; FOI, Follicle-to-Oocyte Index. *p < 0.05, **p < 0.01, ***p < 0.001.

#### 3.3.2 Metabolism of follicular fluid

A total of 836 metabolites in positive and negative ion modes were identified in follicular fluid. These metabolites were primarily including organoheterocyclic compounds, lipids and lipid-like molecules, organic acids and derivatives, and alkaloids and derivatives. After different treatments, there is no significant difference in metabolite composition between the two groups in negative (NEG) ion mode (Supplementary Figures 4A,B). However, when analyzed in positive (POS) ion mode, lipids and lipid-like molecules constituted 89.5% of the composition in A group, in contrast to 70.81% in B group, alkaloids and their derivatives made up 3.16% in A group, as opposed to 13.43% in B group (Supplementary Figures 4C,D).

**Figure 6:**
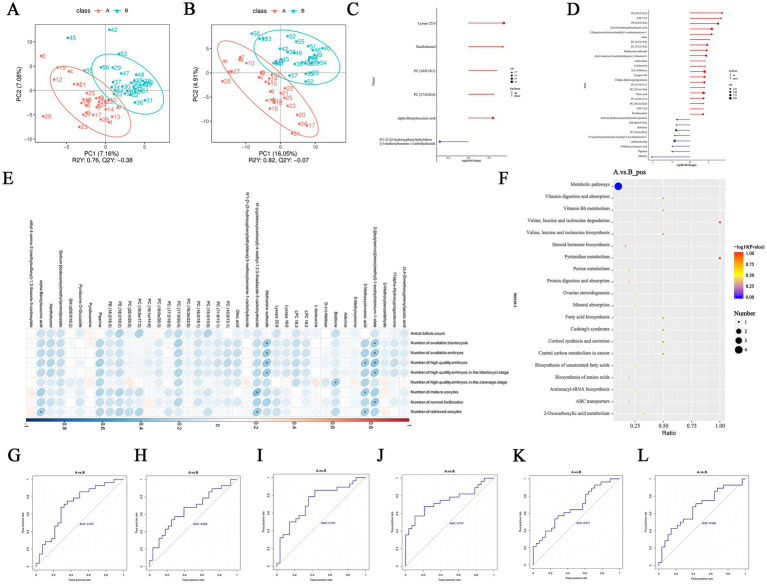
Metabolic changes in follicular fluid of PCOS. **(A,B)** PCOA in positive and negative ions for two groups. **(C,D)** Matchstick plots of differential metabolites for groups A and B. **(E)** The correlation between differential metabolites and oocyte embryo. **(F)** KEGG enrichment diagram of differential metabolites for groups A and B in positive ion. **(G–L)** The receiver operating characteristic (ROC) curve plots of 2-[(butylamino)(imino)methyl]-1-oxohydrazinium-1-olate, 3-Methoxycinnamic acid, alpha-Benzylsuccinic acid, Boldione, Methionine sulfoxide, N’-(cyclohexylcarbonyl)-4-methyl-1,2,3-thiadiazole-5-carbohydrazide.

There were significant differences in the PLS-DA between the two groups (Figures 6A,B). The screening of differential metabolites mainly utilized three parameters: variable importance in projection (VIP), fold change (FC), and *p*-value. The thresholds were set as VIP > 1.0, FC > 1.5 or FC < 0.66, and p-value < 0.05. After acupuncture combined with IVF treatment, there were 6 differential metabolites (5 upregulated and 1 downregulated) in POS ion mode, and 33 differential metabolites (25 upregulated and 8 downregulated) in NEG ion mode (Figures 6C,D; Supplementary Table 9).

Through correlation analysis, it was found that 6 differential metabolites were significantly associated with oocyte and embryo quality (Figure 6E). Among them, boldione, a precursor substance of anabolic steroids, showed a negative correlation with the number of high-quality embryos at the cleavage stage. Methionine sulfoxide (MetO), one of the most easily oxidized amino acids in proteins, was negatively correlated with oocyte and embryo quality. Through KEGG enrichment analysis of differential metabolites between A group and B group (Figure 6F), no enriched pathways were found in the NEG ion mode. In the POS ion mode, the enriched pathways mainly included metabolic pathways, digestion and absorption of amino acids, and biosynthesis of steroid hormones. The receiver operating characteristic (ROC) curve showed that all 6 differential metabolites had certain predictive power (Figures 6G–L).

## 4 Discussion

The study was divided into three components to enhance our understanding of the systemic metabolic characteristics of PCOS and its relationship with gut microbiota, as well as to investigate the potential benefits and mechanisms of action of acupuncture in conjunction with IVF treatment. The first component identified systemic metabolic abnormalities associated with PCOS. Specific bacterial taxa, such as *s_Lachnospiraceae*, *s_Blautia_sp.*, and *g_Escherichia-Shigella*, were correlated with physiological indicators of PCOS, including weight, BMI, waist circumference, WHR, and hormone levels. The second part indicated the regulatory effects of acupuncture combined with IVF on key bacterial species *g_Escherichia-Shigella* and glycolipid metabolism in PCOS. The third part demonstrated the potential advantages of acupuncture combined with IVF treatment in oocyte quality and embryonic developmental potential in PCOS. The key metabolites of follicular fluid, MetO and boldione, were closely related to follicular quality. Although all outcome measures in this study were objective, the open-label design and the possibility of residual or unmeasured confounding nevertheless warrant cautious interpretation of the findings.

### 4.1 Metabolic disturbances related to reproductive endocrinology in PCOS

PCOS has systemic metabolic abnormalities, including obesity (Wang et al., 2023), hyperandrogenism (Livadas et al., 2014), hyperinsulinemia (Greenwood and Huddleston, 2019), lipid metabolism disorders (Saha et al., 2008), and elevated AMH and LH levels (Homburg et al., 2013) in this research and previous studies. Obesity is a common feature in PCOS. While the average BMI in our study did not meet the criteria for obesity, individuals with PCOS exhibited significantly higher weight and BMI compared to the healthy control group, aligning with findings from previous studies (Yu et al., 2022), including one conducted in China (Zhou et al., 2020). In addition, the waist circumference and WHR in PCOS were increased, which linked to reproductive outcomes in previous research (Xia et al., 2024). The 2023 “International Evidence-Based Guidelines for Polycystic Ovary Syndrome” indicated that AMH can serve as an alternative to transvaginal ultrasound for diagnosis PCOS (Teede et al., 2023a), consistent with the clinical manifestations of elevated AMH and LH levels in PCOS (Teede et al., 2023a, 2023b; Guo et al., 2022). In glucose and lipid metabolism, PCOS exhibited higher FINS, HOMA-IR, LDL-C, and APOB levels compared to HC (Guo et al., 2022), with studies linking elevated LDL-C to lower ovulation rates (Cai et al., 2022).

### 4.2 Characteristics and metabolic pathways of gut microbiota in PCOS

Significant differences were observed in gut microbiota composition, abundance, diversity, and KEGG metabolic pathways in PCOS compared to HC., with increased fructose and mannose metabolism (ko00051) (Hanna et al., 2025) and suppressed amino acid metabolism (ko00250) pathways (Yang et al., 2022). Consistent with prior studies, The alpha diversity and beta diversity analyses indicated lower microbial diversity and richness in PCOS (Yu et al., 2022; Zou et al., 2023; da Silva et al., 2024), associated with PCOS pathogenesis (Corrie et al., 2021). *s_Lachnospiraceae*, *s_Blautia_sp.*, and *g_Escherichia-Shigella* are specific gut microbiota of PCOS. The *s_Blautia_sp.* in Chinese PCOS is more abundant than in HC (Yang et al., 2024) and negatively correlated with LH and AMH. *S_Blautia_sp.*, as an anaerobic Gram-positive bacterium commonly found in the gastrointestinal tract of mammals (Murphy et al., 2025), is associated with the production of primary bile acids such as bile acid (Islam et al., 2011; Yu et al., 2022). Bile acid, as a common secondary metabolite, regulates IL-22 to impact ovarian function and hormone secretion and plays an important role in the pathogenesis and treatment of PCOS (Qi et al., 2019). This research found a positive correlation between FBG and *s_Lachnospiraceae*. In the letrozole-induced PCOS mouse model, both *s_Lachnospiraceae* and *s_Blautia_sp.* (Kelley et al., 2016) were increased, which may be closely related to the pathogenesis of PCOS. Both this research and other studies have found the abundance of *g_Escherichia-Shigella* increased in PCOS, with *g_Shigella* potentially making the host more susceptible to metabolic disorders and inflammation, thereby contributing to the pathogenesis of PCOS (Chu et al., 2020; Yu et al., 2022).

Through further metagenomic analysis and KEGG annotation, we found that fructose metabolism (ko00051) in PCOS significantly increased, which is consistent with previous research (Hanna et al., 2025), correlated with FBG. HC exhibited higher energy metabolism pathways, including amino acid metabolism (ko00250) correlated with weight, BMI, and waist circumference, pyrimidine metabolism (ko00240), and general metabolism (ko00760). The previous research found the amino acid metabolism pathway (ko00250) decreased, and energy metabolism pathways such as carbohydrate digestion and absorption are decreased in PCOS compared to HC (Haudum et al., 2020), indicating energy metabolism dysregulation in PCOS.

### 4.3 The positive effect of acupuncture combined with IVF treatment on PCOS metabolism

This study demonstrated that, unlike the observed increase in body weight and BMI following IVF treatment alone, individuals with PCOS who underwent acupuncture in conjunction with IVF treatment experienced a reduction in body weight and BMI. The inter-group comparison of pre- to post-treatment differences was statistically significant (*p* < 0.05). Although no studies have consistently reported universal weight gain during IVF cycles in individuals with PCOS, a high BMI is associated with reduced oocyte quality (Raviv et al., 2020). The B group increased in weight, BMI, hip circumference, APOA1, and FINS after IVF, indicating exacerbated glucose and lipid metabolism disorders. And the A group showed decreased FBG after acupuncture combined with IVF treatment (*p* < 0.05), likely due to acupuncture improving IR by regulating the insulin signaling molecules (Huang et al., 2016). Interestingly, no significant differences in T or TG levels were observed between groups, though both showed increased levels post-treatment, possibly due to ovulation induction drugs like letrozole, which enhance LH and follicle-stimulating hormone, increasing natural T production (Bulow et al., 2022).

### 4.4 The impact of acupuncture combined with IVF treatment on microbiota and metabolic pathways in PCOS

While the post-treatment alpha diversity and beta diversity of the two groups remained unchanged, LEfSe analysis showed the abundance of *g_Escherichia-Shigella* significantly decreased after acupuncture combined with IVF treatment compared to IVF treatment alone (d = 0.307, *p* = 0.017). Furthermore, our team previous studies and other previous researches have indicated that the abundance of *g_Escherichia-Shigella* is significantly elevated in the gut microbiota of PCOS (Liu et al., 2017; Yu et al., 2022) and is associated with the pathogenesis of PCOS, potentially serving as a potential microbial biomarker (Senthilkumar and Arumugam, 2025). The *g_Escherichia-Shigella* is linked to diarrhea (Devanga Ragupathi et al., 2018) and inflammatory bowel diseases (Zhao et al., 2023), inducing gut inflammation, affecting host metabolism and insulin sensitivity, and exacerbating PCOS symptoms, reducing oocyte quality (Corrie et al., 2023). This study found that *g_Escherichia-Shigella* was positively correlated with LDLC and TCH. Previous studies have shown that lipid metabolism improvements coincide with decreased *g_Escherichia-Shigella* abundance (Jin et al., 2021; Li et al., 2024), indicating acupuncture may having a positive effect on lipid metabolism by regulating the abundance of *g_Escherichia-Shigella.* Previous research has reported a negative correlation between the abundance of *g_Escherichia-Shigella* and the level of ghrelin (Liu et al., 2017), a mediator of the brain-gut axis, and a decrease in both ghrelin and peptide YY (PYY) levels in PCOS (Zhang et al., 2019b). These brain-gut axis mediators, including ghrelin and PYY, are believed to regulate appetite and promote mental health in women with PCOS (Konturek et al., 2004; Lang et al., 2015). However, the effects of acupuncture on the gut-brain axis and metabolism in PCOS remain to be investigated. The inflammatory pathways including IL-17 signaling (ko04657), PI3K-Akt signaling (ko04151), and Th17 cell differentiation (ko04659) suppressed in PCOS after the acupuncture combined with IVF treatment, indicating decreased systemic inflammation post-treatment, consistent with prior reports on the effects of acupuncture for PCOS (Cochran et al., 2024).

### 4.5 The positive effect of acupuncture for the quality of oocyte and embryo in PCOS undergoing IVF

The A group had an oocyte maturation rate of 89.4% compared to 87.6% in the B group (*p* > 0.05). It suggested that acupuncture has a positive effect on the quality of oocytes in PCOS undergoing IVF. Mature oocyte rate refers to the proportion of mature oocytes in the number of oocytes retrieved, and is one of the evaluation indicators of oocyte quality. High-quality oocytes are the basis for the development of high-quality embryos, which have better developmental potential and higher pregnancy rates after transfer into the uterus (Gunther et al., 2022). For PCOS with low oocyte maturation rate, increasing oocyte maturation rate can improve the success rate of IVF (Xiang et al., 2021).

### 4.6 Acupuncture combined with IVF may beneficially alter FF metabolism, MetO and boldione representing promising predictors of oocyte quality in PCOS

FF contains essential metabolites for oocyte development (Zhang et al., 2017). Non-targeted metabolomics analysis of FF revealed significant differences, with lipids and lipid-like molecules comprising 89.5% after acupuncture combined with IVF treatment versus 70.81% after IVF treatment only. Lipids and lipid-like molecules are closely related to the quality of oocytes in PCOS (Ding et al., 2022; Zhang et al., 2024). Analysis of the differentially expressed metabolites after intervention revealed that the relative content of boldione was significantly lower. Boldione and MetO were closely related to the development of oocytes in PCOS. Boldione, a precursor to anabolic steroids like T (Tarkowská, 2019), correlated with PCOS symptoms like hirsutism and acne, as well as elevated T levels. Studies have shown that elevated T levels in follicular fluid of PCOS can affect follicular cell development (Eini et al., 2022) and even promote local ovarian inflammation, leading to the pyroptosis of ovarian granulosa cells (Xiang et al., 2023). This study found that boldione is negatively correlated with the number of high-quality embryos at the cleavage stage in PCOS, potentially promoting ovarian inflammation to impact follicular cell development. Recent studies have found that boldione is related to the synthesis pathway of steroid-based drugs (Ke et al., 2024). MetO, a marker of oxidative stress due to methionine oxidation (Cabreiro et al., 2006), was negatively correlated with oocyte and embryo quality (Zhang et al., 2022). MetO had positive causal effects on PCOS risk in a bidirectional mendelian randomization study (Lei et al., 2025). These findings suggested that elevated MetO may induce increased oxidative stress in the ovary, thereby impairing oocyte quality in PCOS. KEGG enrichment analysis revealed the enrichment in steroid hormone biosynthesis, ovarian steroidogenesis, fatty acid biosynthesis, and amino acid biosynthesis. Steroids are involved in follicular development, ovulation, and pregnancy maintenance, as well as in regulating Gn secretion in the systemic circulation (Drummond, 2006). In animal models of anovulation exposed to androgens prenatally, the steroidogenic pathway was impaired in a phenotype-specific manner (Abruzzese et al., 2020), leading to dysfunctional steroid biosynthesis in PCOS. Previous studies also have suggested that steroid biosynthesis is dysfunctional in PCOS (Sander et al., 2011).

### 4.7 Associations between gut microbiota and follicular fluid metabolome alterations induced by acupuncture combined with IVF and their correlations with clinical outcomes in PCOS

Multiple studies have shown that glucose metabolism disorders can lead to mitochondrial dysfunction and abnormal glycolysis in oocytes of PCOS, affecting the switch of oocyte energy metabolism and resulting in a decrease in oocyte maturation rate (Wang and Wu, 2020; Zhang et al., 2022). Dysregulation of lipid metabolism can cause biosynthetic metabolic dysfunction, leading to a decrease in fertilization rate and thus reducing embryo quality in PCOS (Sacks et al., 2018). Acupuncture had a positive effect on balancing glucose and lipid metabolism in PCOS undergoing IVF, regulating the relative abundance of key gut microbiota *g_Escherichia-Shigella* in PCOS, and improving systemic inflammatory metabolic pathways, which may be related to the improvement of oocyte and embryo quality in PCOS by acupuncture. A recent study has also demonstrated that the gut microbiota can ameliorate the reproductive phenotype, inhibit ovarian AMH expression, and modulate key metabolites in PCOS (Li et al., 2025). However, further research is needed to explore this further.

## 5 Conclusion

This study demonstrated that PCOS presents significant differences from HC concerning hormone levels, glucose and lipid metabolism, and the composition, diversity, and abundance of gut microbiota. The combination of acupuncture and IVF was found to regulate the abundance of *g_Escherichia-Shigella* and ameliorate systemic glucose and lipid metabolism disorders. Additionally, this combined treatment exhibited potential benefits in enhancing oocyte quality and embryonic developmental potential, with boldione and MetO identified as key metabolites influencing follicular quality. These findings enhance our understanding of the systemic metabolic characteristics of PCOS and its interaction with gut microbiota, highlighting the potential therapeutic benefits of acupuncture combined with IVF in the treatment of PCOS. This study provides a foundation for further research into PCOS.

## 6 Limitation

Although randomization with allocation concealment, separation of treating practitioners from outcome assessment and analysis, and blinding of assessors and statisticians until study completion were implemented to mitigate the risks associated with the open-label design, participant and practitioner unblinding may have introduced placebo or non-specific effects. As a result, the specificity of the observed effects of acupuncture remains indeterminate, necessitating a cautious interpretation of our findings. A notable limitation of this study is the relatively small sample size, attributable to the limited number of PCOS undergoing IVF/ICSI who provided fully informed and comprehended consent. Future research should involve larger sample sizes and incorporate investigations from multiple reproductive centers to thoroughly examine the metabolic characteristics of PCOS undergoing IVF. Additionally, these studies should assess the clinical efficacy and elucidate the underlying mechanisms of acupuncture as an adjunctive treatment for PCOS undergoing IVF.

## Data Availability

The datasets presented in this study can be found in online repositories. The names of the repository/repositories and accession number(s) can be found at: https://www.ncbi.nlm.nih.gov/, PRJNA1328632.
