# Supplementary material for: Gut microbiota and metabolic dysregulation in polycystic ovary syndrome: effects of acupuncture as an adjunct to in vitro fertilization on gut dysbiosis, metabolism, and oocyte quality
Source: Front Microbiol. 2026 Jan 21;16:1730714. doi: 10.3389/fmicb.2025.1730714 (PMC12868155; doi:10.3389/fmicb.2025.1730714)
Supplement: Supplementary file 1 [file Table_1.docx]

**Gut microbiota and metabolic dysregulation in polycystic ovary syndrome: effects of acupuncture as an adjunct to in vitro fertilization on gut dysbiosis, metabolism, and oocyte quality**

**Supplementary Material**

**Supplementary Table S1. The inclusion and exclusion criteria for PCOS patients**

| **Patients with PCOS** |
| --- |
| **Inclusion criteria:** |
| 1. Age from 21 to 35 |
| 2. Meeting the Rotterdam criteria to diagnose PCOS (Two or more of the following must be conformed): A. clinical and/or biochemical hyperandrogenism (hirsutism and/or an increased testosterone concentration); B. oligo- and/or anovulation (menstrual cycles>35 days and/or the absence of menstruation for at least 3 months); C. polycystic ovaries (assessed through Vaginal ultrasound) |
| 3. IVF or ICSI using GnRH-A ovarian stimulation protocol planned |
| 4. No acupuncture treatment in recent 3 months |
| 5. Not participating in another ongoing clinical study |
| 6. Voluntary participation and signing informed consent |
| **Exclusion criteria:** |
| 1. IVF using other ovarian stimulation protocols than GnRH-A protocol |
| 2. Combined with other endocrine diseases (such as hyperprolactinemia, thyroid disease, etc.) that may affect reproductive function |
| 3. Suffering from uterine malformations (such as unicornuate uterus, bicornuate uterus, etc.) or other diseases that can damage the endometrial cavity (such as adenomyosis, submucosal fibroids, intrauterine adhesions, etc.) |
| 4. Patients or partners with chromosomal abnormalities |
| 5. Patients or partners have severe smoking or alcoholism and other bad habits |
| 6. With bleeding tendency and infection, or suffering from severe allergic diseases, or skin ulcers, scars and other prohibited acupuncture |

**Supplementary Table S2. The inclusion and exclusion criteria for healthy controls**

| **Healthy volunteers** |
| --- |
| **Inclusion criteria:** |
| 1. Age from 21 to 35 |
| 2. Regular menstruation without dysmenorrhea |
| 3. No obvious discomfort |
| 4. Laboratory tests and assessments of each scale were within normal limits |
| 5. Not participating in another ongoing clinical study |
| 6. Voluntary participation and signing informed consent |
| **Exclusion criteria:** |
| 1. Taken antibiotics in the past three months |
| 2. With a history of gastrointestinal surgery |
| 3. With inflammatory bowel disease, irritable bowel syndrome, autoimmune diseases, cancer or other diseases that may affect the gut microbiota |
| 4. With uncontrolled diseases that lead to inflammation |
| 5. With a history of taking probiotics within 1 month |

**Supplementary Table S3. Location of acupoints and manipulation techniques used for the acupuncture + IVF group.**

| **Acupoint** | **Location** | **Manipulation** |
| --- | --- | --- |
| **group 1 in the supine position** | | |
| GV20 (Baihui) | 5 cun directly above the midpoint of the front hairline | Flat insertion to a depth of 0.5 cun-0.8cun |
| GV 24 (Shenting) | 0.5 cun directly above the midpoint of the front hairline | Flat insertion to a depth of 0.5 cun-0.8cun |
| GB 13 (Benshen) | 0.5 cun above the anterior hairline, 3 cun lateral to the Shen Ting acupoint, at the intersection of the inner 2/3 and outer 1/3 of the line connecting Shenting and Touwei acupoints. | Flat insertion to a depth of 0.5 cun-0.8cun |
| CV12 (Zhongwan) | 4 cun to the navel, on the upper abdomen middle | Inserted vertically to a depth of 1.0 cun-1.5cun |
| CV 6 (Qihai) | 1.5 cun below the umbilicus, on the anterior midline | Inserted vertically to a depth of 1.0 cun-2.0 cun |
| CV4 (Guanyuan) | 3 cun below the umbilicus, on the anterior midline | Inserted vertically to a depth of 1.0 cun-2.0 cun |
| ST25 (Tianshu) | Bilateral, On the same level of the umbilicus, and 2 cun lateral to the anterior midline. | Inserted vertically to a depth of 1.0 cun-1.5 cun |
| GB26 (Daimai) | Bilateral, 1.8 cun below to Zhangmen (Zhangmen, below the free end of the 11th floating rib) | Inserted vertically to a depth of 1.0 cun-1.5 cun |
| KI12 (Dahe) | Bilateral, 4 cun below the umbilicus, and 0.5 cun lateral to the lower anterior midline | Inserted vertically to a depth of 0.5 cun-1.0 cun |
| EX-CA1 (Zigong) | Bilateral, 3 cun lateral to the umbilicus, and 4 cun lateral to the lower anterior midline | Oblique insertion to the uterus location direction to a depth of 0.8 cun-1.2 cun |
| ST36 (Zusanli) | Bilateral, 3 cun directly below Dubi, and one fingerbreadth lateral to the anterior border of the tibia (Dubi, in the lateral depression of the patellar ligament, when the knee is flexed) | Inserted vertically to a depth of 1.0 cun-2.0 cun |
| ST40 (Fenglong) | One finger-breadth lateral to Tiaokou, and at the midpoint of the line joining Dubi and the tip of the external malleolus. | Inserted vertically to a depth of 1.0 cun-2.0 cun |
| LR3 (Taichong) | Bilateral, in the depression anterior to the junction of the first and second metatarsal bones | Inserted vertically to a depth of 0.5 cun-0.8 cun |
| **group 2 in the prone position** | | |
| BL23 (Shenshu) | Bilateral, 1.5 cun lateral to the depression below the spinous process of the second lumbar vertebra | Inserted vertically to a depth of 0.5 cun-1.0 cun |
| BL25 (Dachangshu) | Bilateral, 1.5 cun lateral to the depression below the spinous process of the fourth lumbar vertebra | Inserted vertically to a depth of 0.5 cun-1.0 cun |
| BL32 (Ciliao) | Bilateral, in the sacral region, below the anterior superior iliac spine, and precisely at the site of the second sacral foramen | Inserted vertically to a depth of 1.0 cun-1.5 cun |
| BL53 (Zhibian) | Level 4 posterior sacral foramen, 3 cun lateral to the midline of the sacral promontory | Inserted vertically to a depth of 1.0 cun-2.0 cun |
| SP9 (Yinlingquan) | Posteroinferior to the medial condyle of the tibia | Inserted vertically to a depth of 1.0 cun-2.0 cun |
| SP 6 (Sanyinjiao) | Bilateral, Posterior to the mesial border of the tibia, and 3 cun above the tip of the medial malleolus | Inserted vertically to a depth of 1.0 cun-1.5 cun |
| KI3 (Taixi) | Bilateral, on the medial side of the foot, at the depression between the tip of the medial malleolus and the Achilles tendon | Inserted vertically to a depth of 0.5 cun-1.0 cun |

1 cun (approximately 20 mm) is defined as the width of the interphalangeal joint of the patient’s thumb.

Blastocyst is defined as the embryonic stage reached approximately 5-7 days after fertilization , characterized by the following three essential morphological features:

1. A prominent fluid-filled blastocoel cavity that occupies ≥ 50% of the embryo volume (in early stages) and usually completely fills the embryo in more advanced stages;

2. A compact, distinct inner cell mass (ICM) consisting of multiple cells, positioned at one pole;

3. A single layer of flattened trophectoderm (TE) cells forming a cohesive epithelium surrounding the blastocoel and ICM.

The Gardner grading system is the global gold standard for blastocyst morphological assessment.

Format: [Expansion grade (1–6)] + [ICM letter] + [TE letter]

**Supplementary Table S4. Blastocyst expansion and hatching status (Numerical Grade 1–6)**

| **Grade** | **Description** | **Morphology Details** | **Clinical Notes** |
| --- | --- | --- | --- |
| 1 | Early blastocyst | Blastocoel < 50% of embryo volume | Too early; rarely transferred |
| 2 | Early blastocyst | Blastocoel ≥ 50% of embryo volume | Still early; rarely transferred |
| 3 | Full blastocyst | Blastocoel completely fills embryo;  zona not thinned | Minimum for full grading (ICM + TE) |
| 4 | Expanded blastocyst | Blastocoel volume > original embryo;  zona clearly thinned | Common good-quality stage |
| 5 | Hatching blastocyst | Trophectoderm herniating through zona pellucida | Highest implantation potential |
| 6 | Fully hatched blastocyst | Blastocyst completely escaped zona pellucida | Ready for immediate implantation |

**Supplementary Table S5. Inner cell mass (ICM) quality**

| **Grade** | **Description** | **Morphology Details** | **Impact on Live Birth Rate** |
| --- | --- | --- | --- |
| A | Excellent/High quality | Many cells, tightly packed, compact | Highest |
| B | Good/Fair quality | Fewer cells, loosely grouped | Good |
| C | Poor quality | Very few cells, sparse or indistinct | Significantly reduced |

**Supplementary Table S6. Trophectoderm (TE) quality**

| **Grade** | **Description** | **Morphology Details** | **Impact on Ongoing Pregnancy** |
| --- | --- | --- | --- |
| A | Excellent/High quality | Many cells forming cohesive epithelial layer | Best |
| B | Good/Fair quality | Fewer cells, loose epithelium | Acceptable |
| C | Poor quality | Very few cells, sparse or large irregular cells | Markedly reduced |

**Supplementary Table S7. Clinical prioritization of blastocyst grades**

| **Grade** | **Description** | **Morphology Details** | **Impact on Ongoing Pregnancy** |
| --- | --- | --- | --- |
| Highest | 4AA, 5AA, 6AA, 4AB, 5AB | ICM = A + expanded or hatching | 60–75% |
| High | 3AA, 5BA, 4BA, 3AB, 6AB | ICM = A but slightly lower expansion or TE = B | 55–65% |
| Good | 4BB, 5BB, 3BB, 6BB | Classic “double B” – most commonly transferred | 50–60% |
| Acceptable | 4BC, 5BC, 4CB, 5AC, 3BC | One C (usually TE) | 35–50% |
| Lower | Any grade with CC (e.g., 4CC, 5CC) | Both ICM and TE = C | <35% (many clinics discard or freeze for observation) |

**High-quality embryo at blastocyst stage：4AA, 5AA, 4AB, 5AB**

**A high-quality cleavage-stage embryo** is defined as one that meets the following key morphological and dynamic criteria simultaneously:

1. Cell number: Exactly 8 cells on Day 3 (7-9 cells still high-priority); 4 cells on Day 2.

Fragmentation: < 10% by volume.

1. Blastomere symmetry/size: Evenly sized blastomeres (no cell diameter deviation ≥ 25%); stage-appropriate size; tetrahedral (3D) arrangement preferred over planar.
2. Multinucleation: Absent.
3. Cleavage pattern: Normal sequential cleavage; no abnormal patterns.
4. Compaction: Initiation from ≥ 8-cell stage.
5. Additional desirable features (especially with TLT): Early first cleavage (<25-27 h post-insemination), absence of vacuoles/granularity, no cytoplasmic strings or severe dysmorphisms from oocyte stage.

**Supplementary Table S8. Baseline characteristics of the participants**

| **Characteristics** | **acupuncture + IVF**  **(A) group (n=28)** | **IVF**  **(B) group (n=28)** | **P-value** |
| --- | --- | --- | --- |
| Age (years), (mean±SD) | 29.86±4.025 | 29.36±3.347 | 0.793 |
| Weight (kg), M (IQR) | 56.650(7.8) | 55.000(13.9) | 0.793 |
| BMI (kg/m2), M (IQR) | 22.730(4.49) | 22.350(4.60) | 0.967 |
| Waist circumference (cm), (mean±SD) | 79.381±7.192 | 78.757±10.136 | 0.792 |
| Hip circumference (cm), (mean±SD) | 93.845±5.111 | 93.329±6.527 | 0.743 |
| Waist to hip ratio, (mean±SD) | 0.844±0.047 | 0.842±0.061 | 0.903 |
| AMH (ng/ml), (mean±SD) | 9.170±4.566 | 9.192±4.006 | 0.985 |
| AFC (number), M (IQR) | 31(18) | 29(10) | 0.869 |
| Duration of infertility (years), M (IQR) | 4.00(4) | 2.00(2) | 0.103 |
| Combined with infertility causes |  |  |  |
| Male factor | 3/28(10.7%) | 3/28(10.7%) | 1.000 |
| Tubal | 8/28(28.6%) | 9/28(32.1%) | 0.771 |
| No. of prior IVF cycles |  |  |  |
| 0 | 19/28(67.9%) | 22/28(78.6%) | 0.365 |
| 1 | 6/28(21.4%) | 4/28(14.3%) | 0.727 |
| ≥2 | 3/28(10.7%) | 2/28(7.1%) | 1.000 |
| Serum levels |  |  |  |
| Total testosterone (ng/dL), M (IQR)  (mean±SD) | 53.575(31.80) | 45.320(24.14) | 0.481 |
| LH (mIU/mL), M (IQR) | 6.815(4.297) | 6.035(5.185) | 0.799 |
| FSH (mIU/mL), M (IQR) | 5.715(2.90) | 5.780(2.38) | 0.372 |
| PRL (uIU/mL), M (IQR) | 283.430(199.75) | 232.130(158.46) | 0.793 |
| E2 (pg/mL), M (IQR) | 28.575(14.56) | 27.985(20.25) | 0.876 |
| P (ng/ml), M (IQR) | 0.435(0.37) | 0.465(0.35) | 0.694 |
| Glycometabolism |  |  |  |
| FBG (mmol/L) (mean±SD) | 5.236±0.335 | 5.199±0.344 | 0.684 |
| FINS (mIU/L), M (IQR) | 6.360(4.93) | 8.720(7.24) | 0.088 |
| HOMA-IR, M (IQR) | 1.510(1.16) | 2.125(1.75) | 0.088 |
| Lipid Metabolism |  |  |  |
| TCH (mmol/L) (mean±SD) | 4.598±0.625 | 4.325±0.551 | 0.090 |
| TG (mmol/L), M (IQR) | 1.375(0.93) | 1.445(0.75) | 0.974 |
| HDL-C(mmol/L), M (IQR) | 1.255(0.46) | 1.125(0.25) | 0.081 |
| LDL-C(mmol/L), (mean±SD) | 2.973±0.564 | 2.746±0.546 | 0.132 |
| APOA1(g/L), M (IQR) | 1.400(0.26) | 1.245(0.35) | 0.090 |
| APOB(g/L), (mean±SD) | 0.856±0.156 | 0.826±0.170 | 0.495 |
| Race, n (%) |  |  | 1.000 |
| Han | 26/28(92.9%) | 27/28(96.4%) |  |
| Minorities | 2/28(7.1%) | 1/28(3.6%) |  |
| Current drinker | 3/28(10.7%) | 1/28(3.6%) | 0.604 |
| Current smoker | 1/28(3.6%) | 1/28(3.6%) | 1.000 |

**Supplementary Table S9. Differentially expressed metabolites**

| **Compound_ID** | **Name** | **FC** | **Pvalue** | **VIP** | **Up.**  **Down** |
| --- | --- | --- | --- | --- | --- |
| Com_13759_neg | Lysops 22:6 | 1.813 | 0.002 | 2.328 | up |
| Com_5818_neg | alpha-Benzylsuccinic acid | 1.513 | 0.005 | 2.194 | up |
| Com_2340_neg | N'1-[1-(2-hydroxyphenyl)ethylidene]-3-methoxybenzene-1-carbohydrazide | 0.618 | 0.015 | 2.010 | down |
| Com_9628_neg | Xanthohumol | 1.779 | 0.027 | 1.746 | up |
| Com_201_neg | PC (16:0/18:1) | 1.599 | 0.043 | 1.546 | up |
| Com_2297_neg | PC (17:0/20:4) | 1.535 | 0.045 | 1.530 | up |
| Com_14897_pos | Lysopa 16:0 | 1.726 | 0.000 | 2.035 | up |
| Com_7318_pos | Oleic acid | 1.657 | 0.000 | 1.975 | up |
| Com_19889_pos | (3,4-Dimethoxyphenyl)acetic acid | 2.249 | 0.001 | 1.868 | up |
| Com_3352_pos | PE (18:2/18:2) | 2.837 | 0.001 | 1.595 | up |
| Com_139_pos | ethyl 4-amino-2-(methylsulfanyl)-1,3-thiazole-5-carboxylate | 1.823 | 0.001 | 1.584 | up |
| Com_22868_pos | Boldione | 0.601 | 0.001 | 1.796 | down |
| Com_7727_pos | 5-Methylcytosine | 1.569 | 0.001 | 1.736 | up |
| Com_2542_pos | PC (17:2/18:5) | 1.905 | 0.002 | 1.495 | up |
| Com_8979_pos | 2-Methoxybenzaldehyde | 1.585 | 0.002 | 1.443 | up |
| Com_4491_pos | Pyridoxamine | 1.634 | 0.003 | 1.408 | up |
| Com_4988_pos | PC (10:0/13:1) | 1.655 | 0.009 | 1.427 | up |
| Com_6762_pos | 2-[(butylamino)(imino)methyl]-1-oxohydrazinium-1-olate | 2.235 | 0.009 | 1.381 | up |
| Com_8283_pos | 17alpha-Hydroxyprogesterone | 1.714 | 0.014 | 1.486 | up |
| Com_339_pos | SM (d26:0/16:2) | 0.606 | 0.015 | 1.161 | down |
| Com_4149_pos | PE (18:2/18:3) | 3.278 | 0.016 | 1.350 | up |
| Com_3559_pos | PC (18:1e/14:0) | 1.698 | 0.019 | 1.108 | up |
| Com_184_pos | LPC 18:3 | 3.179 | 0.020 | 1.189 | up |
| Com_11255_pos | Methionine sulfoxide | 1.860 | 0.022 | 1.420 | up |
| Com_15044_pos | PC (16:2e/22:6) | 1.644 | 0.025 | 1.122 | up |
| Com_2961_pos | L-Isoleucine | 1.777 | 0.029 | 1.052 | up |
| Com_3396_pos | Sodium [dodecanoyl(methyl)amino]acetate | 0.621 | 0.029 | 1.277 | down |
| Com_7071_pos | Pyridoxine O-Glucoside | 1.605 | 0.031 | 1.050 | up |
| Com_2282_pos | Adenine | 0.244 | 0.033 | 1.162 | down |
| Com_6967_pos | PC (18:5e/17:2) | 1.568 | 0.034 | 1.012 | up |
| Com_3695_pos | 3-Methoxycinnamic acid | 0.523 | 0.035 | 1.116 | down |
| Com_10861_pos | PC (18:0e/20:3) | 0.570 | 0.035 | 1.152 | down |
| Com_12733_pos | N'-(cyclohexylcarbonyl)-4-methyl-1,2,3-thiadiazole-5-carbohydrazide | 0.568 | 0.036 | 2.006 | down |
| Com_7566_pos | PC (11:0/13:1) | 1.708 | 0.038 | 1.484 | up |
| Com_793_pos | D-(+)-Maltose | 1.756 | 0.039 | 1.089 | up |
| Com_1050_pos | PC (15:0/15:0) | 2.040 | 0.039 | 1.094 | up |
| Com_5337_pos | PC (19:2/20:5) | 1.542 | 0.039 | 1.010 | up |
| Com_141_pos | Piperine | 0.519 | 0.042 | 1.210 | down |
| Com_4055_pos | LPC 16:2 | 1.643 | 0.043 | 1.044 | up |

**
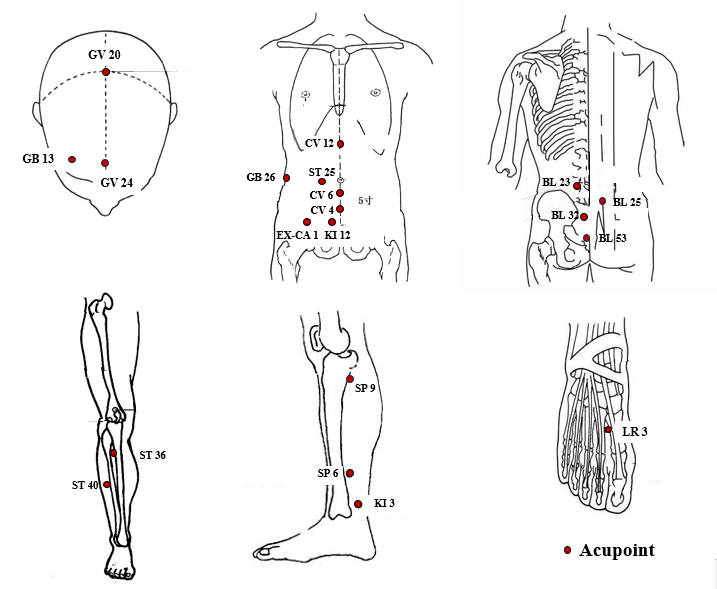
**

**Supplementary Figure 1 Locations of acupoints**

**
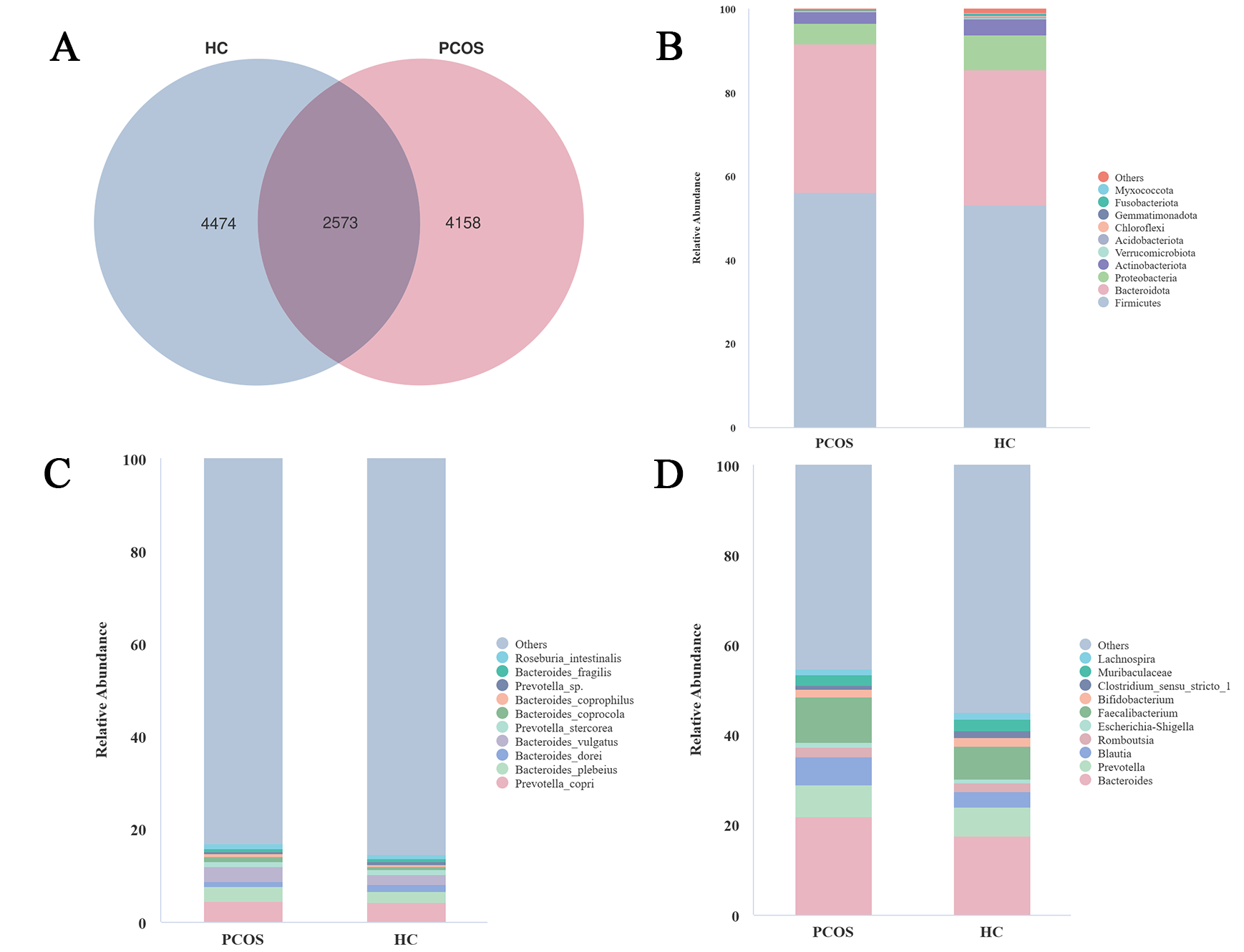
**

**Supplementary Figure 2 The composition of the microbiota in PCOS and HC**

**
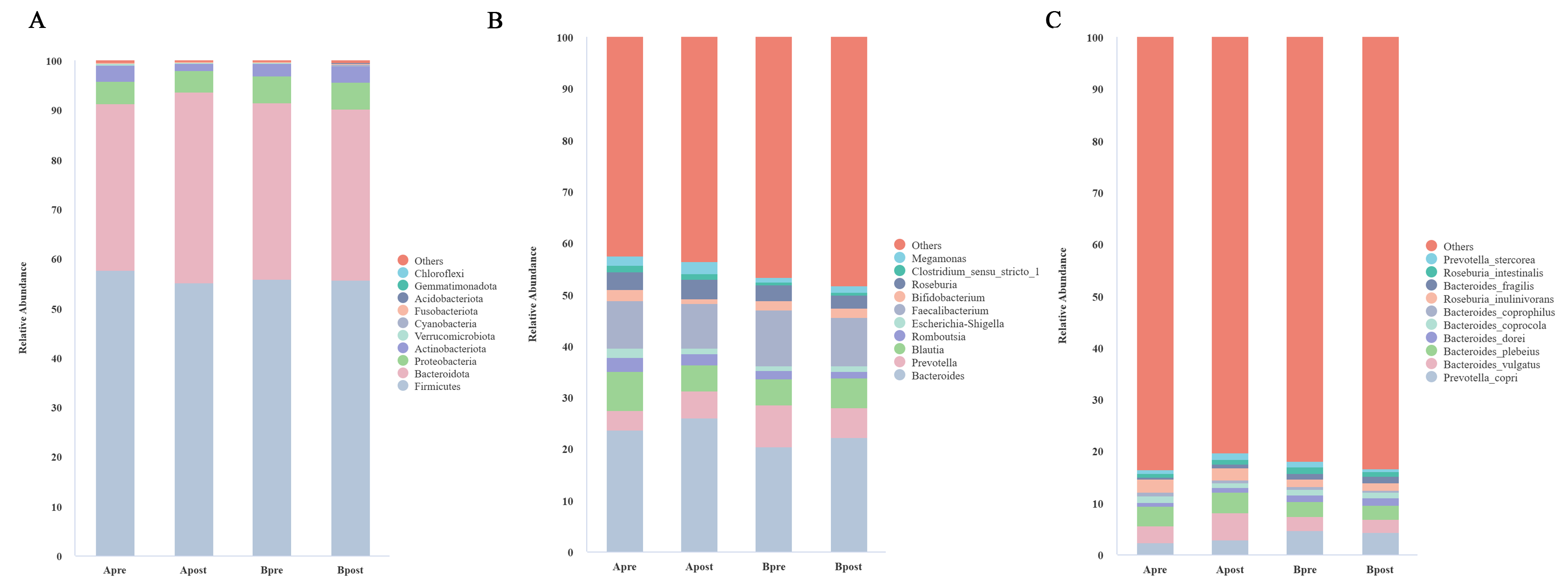
**

**Supplementary Figure 3 The composition of the microbiota between Group A and Group B before and after treatment**

**
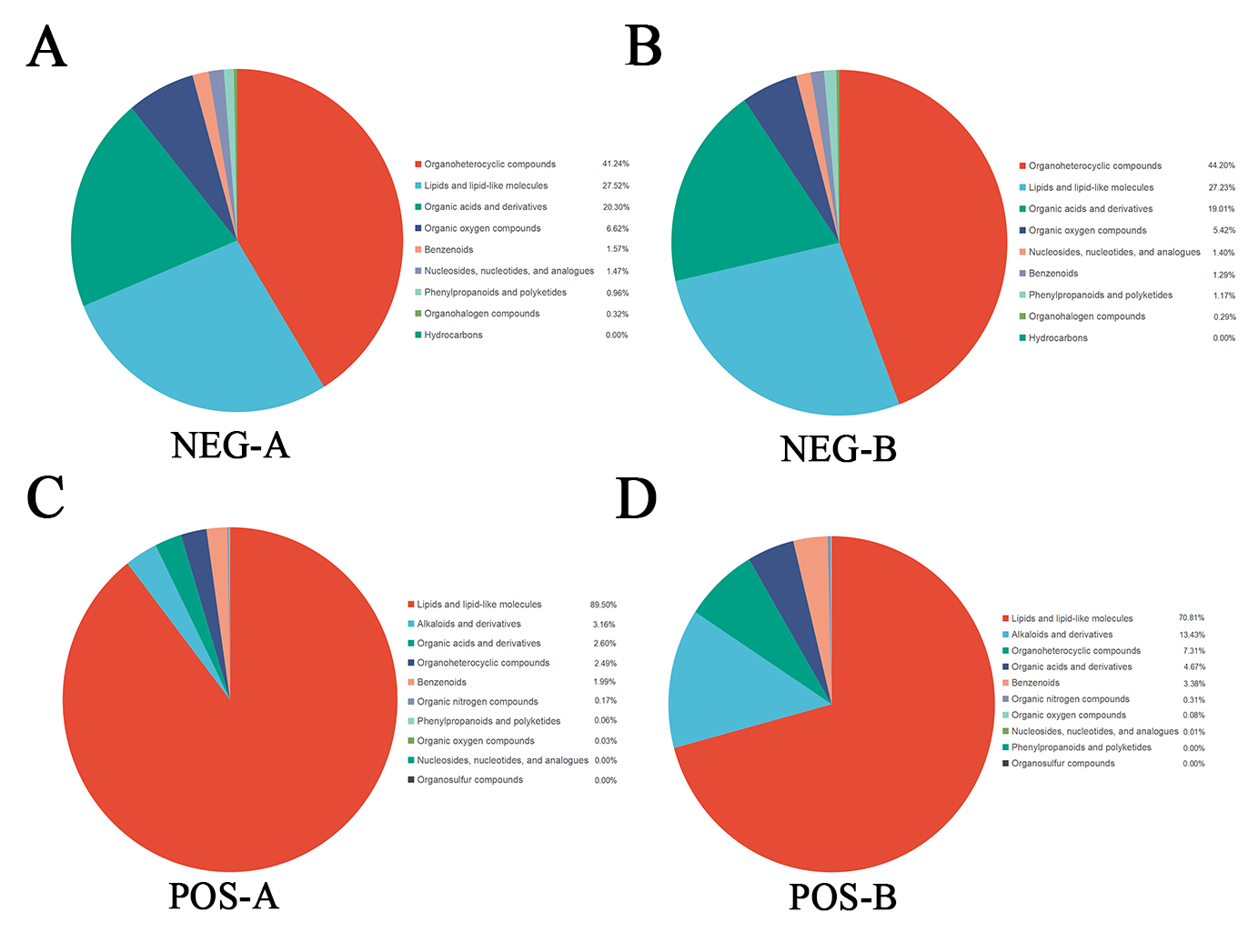
**

**Supplementary Figure 4 Composition of CLASS1 in positive and negative ion modes**
